# Can Early Intervention Improve Maternal Well-Being? Evidence from a Randomized Controlled Trial

**DOI:** 10.1371/journal.pone.0169829

**Published:** 2017-01-17

**Authors:** Orla Doyle, Liam Delaney, Christine O’Farrelly, Nick Fitzpatrick, Michael Daly

**Affiliations:** 1 UCD School of Economics & UCD Geary Institute for Public Policy, University College Dublin, Belfield, Dublin 4, Ireland; 2 Behavioural Science Centre, Stirling Management School, Stirling University, United Kingdom & UCD Geary Institute for Public Policy, University College Dublin, Belfield, Dublin 4, Ireland; 3 Centre for Mental Health, Imperial College London, Commonwealth Building, Hammersmith Hospital Campus, Du Cane Road, London, United Kingdom; 4 Frontier Economics, 71 High Holborn, London, United Kingdom; TNO, NETHERLANDS

## Abstract

**Objective:**

This study estimates the effect of a targeted early childhood intervention program on global and experienced measures of maternal well-being utilizing a randomized controlled trial design. The primary aim of the intervention is to improve children’s school readiness skills by working directly with parents to improve their knowledge of child development and parenting behavior. One potential externality of the program is well-being benefits for parents given its direct focus on improving parental coping, self-efficacy, and problem solving skills, as well as generating an indirect effect on parental well-being by targeting child developmental problems.

**Methods:**

Participants from a socio-economically disadvantaged community are randomly assigned during pregnancy to an intensive 5-year home visiting parenting program or a control group. We estimate and compare treatment effects on multiple measures of global and experienced well-being using permutation testing to account for small sample size and a stepdown procedure to account for multiple testing.

**Results:**

The intervention has no impact on global well-being as measured by life satisfaction and parenting stress or experienced negative affect using episodic reports derived from the Day Reconstruction Method (DRM). Treatment effects are observed on measures of experienced positive affect derived from the DRM and a measure of mood yesterday.

**Conclusion:**

The limited treatment effects suggest that early intervention programs may produce some improvements in experienced positive well-being, but no effects on negative aspects of well-being. Different findings across measures may result as experienced measures of well-being avoid the cognitive biases that impinge upon global assessments.

## Introduction

Understanding the impact of targeted early intervention policies on the life-long development of children is an increasingly important focus of modern policymakers. One potential externality of such interventions is welfare improvements for parents, particularly for policies that target parenting and coping skills. Such benefits may yield value both directly, through their immediate impact on parental utility, and indirectly, through improvements in child health and development. Understanding how to quantify these benefits is essential for providing a full account of the costs and benefits of early intervention policies.

The identification of the utility effects of public policies is frequently hampered by non-experimental designs which limit inferences regarding causality. Randomized controlled trials are widely considered the most robust means of determining impact [1], yet few experimental evaluations incorporate comprehensive measures of utility into estimates of treatment effects. Global well-being measures are increasingly used as direct measure of utility and are based on retrospective assessments of evaluative (e.g. life satisfaction) and hedonic (e.g. happiness) well-being. More recently, studies have argued for a more disaggregated approach where experienced utility is measured at the level of the day or even in real-time e.g. [2, 3]. To date, few studies have used these utility flow measures to evaluate public policies, including targeted intervention programs.

In this paper, we report findings on the impact of an early intervention program on the well-being of mothers in a disadvantaged area of Ireland. Our paper adds to the literature by exploiting a randomized controlled trial in which participants are assigned to an intensive five-year home visiting parenting program or a control group that receives low level supports common to both groups. The primary aim of the program is to improve children’s school readiness skills by working directly with parents to improve their knowledge of child development and parenting behavior. Thus, one potential externality of the program is well-being effects for parents given its focus on improving parental coping, self-efficacy, and problem solving skills. In particular, the logic model underlying the program is based on the assumption that promoting change in parents’ knowledge, attitudes, and well-being would mediate gains for children by increasing parenthood enjoyment and developing secure parent-child relationships [4]. Previous studies on the impact of this program up to 36 months of age identified a number of effects on the primary outcomes of the trial, namely, children’s cognitive, behavioral, and physical health [5, 6]. It is possible that such improvements in child outcomes may be mediated by improvements in parental outcomes, or improvements in child outcomes may lead to improvements in parental outcomes. The objective of this paper is to test for program effects on parental well-being, a secondary outcome of the trial, using a novel combination of methods.

The study is the first to examine the impact of a policy intervention on measures of both experienced and global well-being using an experimental design. This distinction between experienced and global well-being has been described as reflecting the difference between “living life” and “thinking about life” [7]. In this study, global well-being is captured using measures of life satisfaction and a standardized measure of parenting stress. Experienced well-being is captured using daily reports of average, positive, and negative affect derived from the Day Reconstruction Method (DRM) and a measure of mood yesterday. As the DRM incorporates time use data, it allows us to measure parental well-being during times spent with and without the target child. This is particularly relevant given the ambiguity of the effect of children on parental well-being, an issue that is complicated by selection into parenthood [8, 9]. Thus, measuring well-being at multiple points of the day may help to improve understanding about the causal relationship between children and parental well-being. Time use data also allows us to determine whether any identified treatment effects are driven by differences in parents’ daily activities.

Utilizing previously developed methodology [10], we employ permutation testing to address issues relating to the small sample size used and, as a robustness test, we apply a stepdown procedure to mitigate the likelihood of accepting a false positive due to multiple hypothesis testing. Finally, we estimate unconditional models, in addition to conditional models, which allow us to control for any baseline imbalance between the groups.

Overall, we find limited evidence that the program improves maternal well-being, however we do identify a treatment effect on experienced reports of happiness across episodes of the study day as measured by the DRM. In most specifications, this applies to episodes both with and without the target child. We also find a treatment effect on an experienced measure of mood yesterday, yet not during periods when participants are with their child(ren). Consistent with the early intervention literature, the program has no impact on negative aspects of well-being, including experienced negative affect and a global standardized measure of parenting stress. In addition, while higher proportions of the treatment group report being satisfied with their lives compared to the control group, these differences do not reach statistical significance. We also find no differences between the treatment and control groups in time use across the study day concerning the amount of time or types of activities mothers engage in with their child.

The paper is structured as follows. The next section outlines conceptual issues involved in measuring well-being and their relevance for the evaluation of early intervention programs. This is followed by a description of the intervention under investigation and the well-being measures employed. Next, we outline our empirical model and statistical methods before presenting the results. Finally, we discuss the findings and conclude.

## Background and Literature

### Well-being and evaluation of public policy

The use of well-being measures in public policy has been widely debated in recent years [11]. Concerns regarding an overreliance on financial measures of utility have led to calls for global well-being measures to be incorporated into national progress indicators e.g. [12, 13, 14, 15]. There is also a growing interest in using well-being measures to evaluate public goods and policies [16, 17, 18, 19, 20]. However, one issue with this approach is the identification of causal effects, and while instrumental variable estimates or exploiting fine-grained exogenous variation in the provision of the good e.g. [21], can be used, these methods require restrictive assumptions. Thus, it is becoming increasingly common to pilot test provision of public goods using random assignment [22, 23].

### Maternal welfare and early intervention p

Regarding policies which aim to boost children’s skills, recent studies using random assignment have focused on targeted early intervention programs [24, 10, 25]. Less work, however, has examined the effect of these interventions on the welfare of parents. While such effects may exist, it is difficult to hypothesise the likely direction of the effect. For example, their impact on parental consumption may be ambiguous if there are substitution effects whereby parents reduce their employment in order to spend more time with their children. Consequently, measuring parental welfare directly may prove more informative regarding the utility effects of early intervention programs.

Home visiting programs (HVPs) are a common form of early intervention that aim to mediate gains for children by working directly with parents [26]. Such programs may result in improved parental well-being as they typically target maternal health, encourage parents to adopt sensitive, responsive, and consistent parenting behaviors, and assist in family planning and the pursuit of education and employment opportunities [27]. Despite this conceptual premise, HVP studies do not always examine outcomes for parents and children or explicitly test these pathways [26]. Nonetheless, meta-analytic findings suggests that the effects for parents are concentrated on parenting behaviors, attitudes, and skills [28, 29]. There is also evidence, albeit less consistent, for improvements in parental life course outcomes [28, 29].

Less is known about the impact of HVPs on parental psychological well-being. On the one hand, HVPs may improve well-being directly through improved maternal coping, problem solving, and self-efficacy skills, and through the therapeutic relationship with the home visitor, and indirectly through the reduction of child behavioral problems, parent-child conflict, changes in parental health behaviors, and increased social support–although evidence for these outcomes is mixed e.g., [30]. Alternatively, drawing on the family investment theory [31], HVPs may have deleterious effects on well-being if the intervention promotes substantial parental investment in the child which comes at a cost of increased parental time, effort, and emotional outlays in the short-run, with the expectation that parental utility will increase in the long run.

Research in the HVP field has focused predominantly on global measures of negative affect given the burden that stress and depression exert on parent functioning and the subsequent consequences for child well-being e.g., [32, 33, 34]. Yet, a systematic review found that HVPs are not sufficiently powerful, in and of themselves, to substantially mitigate depression as measured by standardized self-report instruments [35]. Equally, HVPs tend not to be effective in reducing parent-reported levels of stress [29]. Comparatively fewer studies have examined the impact of HVPs on positive aspects of parental well-being such as self-efficacy and self-esteem. Theories of self-efficacy, which link people’s beliefs about their capabilities to their subsequent motivation, behavior, and well-being [36], are central to many HVPs [27]. Studies that have examined positive aspects of well-being are inconclusive [37, 38], and have yet to be subject to systematic review. The evidence to date suggests that it may be easier for HVPs to alter parenting behaviors than emotional states [39].

### Global versus experienced measures of well-being

A critical issue for evaluations of public policies, including early intervention programs, is how well-being should be measured [40, 41]. A growing literature has emerged on the use of global retrospective measures of well-being, such as evaluations of life satisfaction and accounts of happiness. These measures have the advantage of providing information on appraisal of circumstances and feelings about them; however debate exists regarding their consistency. A number of studies have documented how immediate mood and context can bias retrospective evaluations, and have argued that the act of thinking about such quantities may focus individuals on aspects of their life that are not crucial to their actual well-being (e.g., [42]). Furthermore, retrospective happiness accounts tend not to accurately represent experience as such accounts are overly influenced by intense or recent experiences [3]. In addition, people may simply fail to accurately recall their well-being over extended periods of several days or weeks, introducing error into well-being estimates.

It has been argued that experienced utility is a more reliable measure of well-being as it directly captures emotional experiences in real time [2]. The experience sampling approach collects information on respondents’ self-reported emotional responses to their daily experiences at specific points during a day using electronic devices as prompts [43]. It has been widely applied in clinical psychology and psychiatry studies e.g., [44, 45, 46, 47, 48, 49]

The use of the DRM has been proposed as an alternative means of recording fluctuations in experienced well-being in a less burdensome manner [3]. The DRM is completed in a single session during which respondents divide the previous day into discrete episodes which are then rated across several positive and negative affective states. Compared with experience sampling, the DRM has the advantage of eliciting events over an entire day without interfering with the day’s activities. The DRM has been used in a variety of non-experimental settings including measuring time use and emotional well-being among the unemployed [40, 50], examining individuals with optimal mental health [51], and studying women during the transition to motherhood [52].

Another important distinction when measuring well-being concerns positive and negative affect, which have been shown to represent different dimensions of well-being with distinct correlates. For example, negative affect (including feelings of stress, anxiety, anger, and impatience) is traditionally associated with health issues, whereas positive affect (including feelings of happiness, calm, focus, and control) is associated with social engagement [53, 54, 55]. An advantage of the DRM is its ability to elicit ratings of both positive and negative affect.

One potential concern when using the DRM is that respondents may not accurately recall emotions experienced the previous day. Several studies have examined this issue by comparing DRM ratings with ratings provided in real time using experienced sampling methods, and all find a reasonably high degree of convergence [45, 56, 3, 57, 58]. Furthermore, a positive correlation between DRM measures of negative affect and fluctuations in heart rate, an objective indicator of psychological stress, has been found [59]. See [60] for a critical review of DRM research.

Although the DRM is less burdensome than experienced sampling, it nonetheless requires participant effort [61]. Consequently, interest has developed in less intensive measures of experienced well-being that are still robust to cognitive biases which affect global measures. One practical alternative is a measure of mood yesterday which requires respondents to provide an overall appraisal of their emotional states across the course of the previous day. Although these measures have been incorporated in some large scale social surveys, evidence is still needed to endorse their value as a viable proxy for more intensive measures of experienced affect [62].

## Material and Methods

### Experimental set-up

The RCT was registered with the International Standard Randomised Controlled Trial Number (ISRCTN) register, (unique identifier ISRCTN04631728—The evaluation of the Preparing For Life early childhood intervention programme, http://www.controlled-trials.com/ISRCTN04631728). As the program is a community-based intervention targeting school readiness skills rather than a clinical trial examining health outcomes, the trial was registered post-recruitment rather than prospectively. All study procedures were approved by the UCD Human Research Ethics Committee, the Rotunda Hospital Ethics Committee, and the National Maternity Hospital Ethics Committee, and was conducted and reported in conformity with CONSORT guidelines (see S1 Protocol). All participants gave written informed consent before randomization. Written informed consent for those under the age of 18 was provided by their parents/guardians. Information on the design of the trial has been published elsewhere [63] (also see S2 Protocol).

The original study enrolled pregnant women from a suburban community in Dublin, Ireland, which had above national average rates of unemployment, school dropout, lone parent households, and public housing. The inclusion criteria included all pregnant women living in the catchment area during the recruitment period, regardless of parity. There were no exclusion criteria. This within-community universal approach was adopted to avoid the stigmatization which may arise in programs with highly selective inclusion criteria. Participation was voluntary and recruitment took place between the 29^th^ of January 2008 and the 4^th^ of August 2010 through two maternity hospitals and in the community. Recruitment and randomization were conducted by the program recruitment officer.

The sample size was calculated based on a small effect size (ES, standardized difference between group means) for child school readiness skills as identified by a previous meta-analytic study of home visiting programs [29]. Specifically, a mean difference between the treatment and control groups of between 2 and 5 points (depending on the study included in the meta-analysis) on standardized cognitive development scores (average standardized ES = 0.184) was expected. Given this effect size, in order to power the study at the 80% level, based on an alpha level of .05 using a two-tailed t-test, a sample size of approximately 117 in both groups was required. In total, 233 participants were recruited and a computerised unconditional probability randomization procedure, with no stratification or block techniques, assigned 115 participants to the treatment group and 118 to the control group. To ensure randomization was not compromised, the computerized procedure generated an automatic email which was sent to the program manager and the principal investigator and included the participant’s assignment condition and identification code. Attempts to reassign participants would trigger a second email highlighting any intentional subversion of the randomization process.

The population based recruitment rate was 52% based on the number of live births in the community during the recruitment window. A further 22% of eligible participants were not contactable and a further 26% met the program recruiter or made contact but did not join the program. To identify whether there are systematic differences between eligible participants and eligible non-participants, a socio-demographic profile survey was conducted with a sample of eligible non-participants (n = 102) when their children were 4 years old. An analysis of these data indicated that the eligible non-participants were of a slightly higher socioeconomic status than the participants who joined the program. This suggests that the program was effective in targeting the families most in need of intervention.

There were no statistically significant differences between the original treatment and control groups on 90.5% (114/126) of baseline variables, suggesting the randomization procedure was successful [63].

The treatment included the Preparing for Life (PFL) HVP [4] and the Triple P Positive Parenting Program [64]. The treatment aims to improve the health and development of children by intervening during pregnancy and working with families until the children start school at age 4/5. The program was developed in response to evidence that children from the catchment area were lagging behind their peers in terms of cognitive and non-cognitive skills at school entry [65]. PFL is a manualized program which is grounded in the theories of human attachment [66], socio-ecological development [67], and social-learning [36].

#### Treatment

The treatment prescribes twice monthly home visits, lasting approximately one hour, delivered by mentors from a cross-section of professional backgrounds including education, social care, and youth studies. Mentors received extensive training prior to program implementation and monthly supervision thereafter. Each family is assigned the same mentor over the course of the treatment where possible. The home visits are tailored based on the age of the child and the needs of the family and are guided by a set of Tip Sheets presenting best-practice information on pregnancy, parenting, and child health and development.

This study refers to the impact of the treatment on a secondary outcome, maternal well-being, and includes participants who were engaged with the program for at least two and a half years. The program is anticipated to have an impact on well-being due to the nature of the mentor-mother relationship and the supports provided. Specifically, the mentors support mothers by building a strong relationship with them and helping them to improve their parenting and problem solving skills using role modelling, coaching, discussion, encouragement, and feedback. In addition, a number of Tip Sheets delivered between pregnancy and the child’s second birthday focus on maternal personal and social well-being, including the mother’s relationship with the father, social support, support services available in the community, self-care, exercise, and postnatal depression. For example, one Tip Sheet provides information on the prevalence and symptoms of postnatal depression, while a Tip Sheet on self-care suggests that mothers reward themselves by relaxing and doing something that makes them feel good.

The treatment group are also invited to participate in an additional parenting course (Triple P Positive Parenting Program) [68] when their children are between 2 and 3 years old. Triple P promotes healthy parenting practices and positive parent-child attachment. Meta-analysis of Triple P has demonstrated positive effects for parenting practices and children’s social, emotional, and behavioral outcomes [68]. The majority of treatment participants took part in Group Triple P which consists of five 2-hour group discussion sessions and three individual phone calls facilitated by the mentors.

#### Common supports

Both the treatment and control groups receive some common supports including developmental materials and book packs. Both groups are also encouraged to attend public health workshops on stress management and healthy eating which are already available to the wider community, however relatively few members of either group attend these sessions. The control group also has access to a support worker who can help them avail of community services if needed, while this function is provided by the mentors for the treatment group.

### Participants

**Fig 1:**
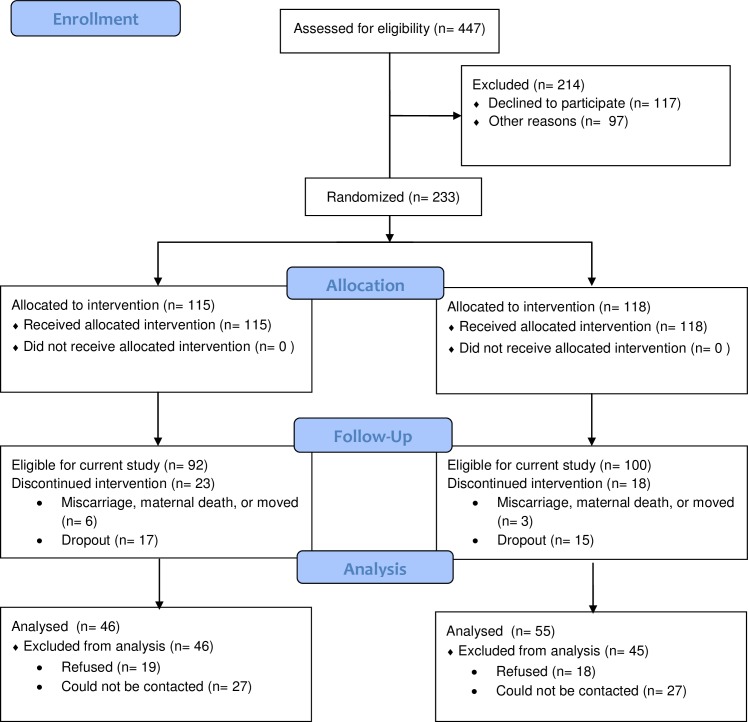
CONSORT Flow Diagram.

Of the original 233 participants, 192 were eligible to participate in the well-being sub-study as they had not voluntarily or involuntarily dropped out of the original study at the time of data collection. 32 participants (treatment = 17; control = 15) voluntarily dropped out and a further 9 (treatment = 6; control = 3) involuntarily dropped out due to miscarriage, maternal death, child death, or moved out of the catchment area at the time of data collection. Fig 1 depicts the CONSORT diagram for participants in the original trial and the present sub-study. Mothers were invited to take part in the sub-study by telephone, and a flyer was sent to those who could not be reached. The study was described as “A Day in the Life of a Parent”, the goal of which was to collect information on parents’ daily lives and to learn about the different emotions parents experience during a typical day. Of the 192 target participants, 101 (treatment = 46; control = 55) took part in the sub-study, 34 refused, 2 agreed but did not participate, and 54 could not be reached by telephone, text, or letter. Participants were at various stages in the program when they participated in the sub-study; the youngest child was 24.6 months and the oldest child was 62.5 months old. Thus, program duration differs for each participant as data collection was conducted over a one year period.

In order to test for selection into the sub-study, we compare those who participated to those who did not on 48 baseline measures of socio-demographics, health, parenting, and psychometrics. Participants who chose to take part in the sub-study did not differ from those who did not on 96% of the baseline characteristics (46/48) using two-tailed tests with a 10% cut-off for significance. Significant differences on 2 (4%) measures indicated that mothers in the sub-study were more open (as per the Ten Item Personality Index (TIPI) [69]), and more likely to have their activity impaired by illness. Importantly, there is no selection into the sub-study based on treatment status as 46% of the treatment group participated in the sub-study and 54% of the control group (p = 0.287). This suggests that there was no systematic selection into the sub-study based on a wide range of observable characteristics.

S1 Table presents descriptive statistics on the participating sample for a selection of the baseline variables disaggregated by treatment status. On average, mothers were between 25 and 26 years old and had one non-PFL child. Approximately half of participants were first time mothers, over 55% lived in public housing, and approximately 40% had not completed second level education and identified themselves as being unemployed. A significantly higher proportion of treatment mothers had a boy as their PFL target child (48%) than control mothers (31%).

An analysis examining differences between the treatment and control groups who participated in the sub-study found that the groups do not differ on 92% (44/48) of baseline measures. This suggests that the randomization assumption is still valid. Significant differences on the 4 (8%) measures indicate that the treatment group were less likely to exercise, had lower self-efficacy scores [(as per the Pearlin Self Efficacy Scale [70]) and emotional attachment scores (as per the Vulnerable Attachment Style Questionnaire (VASQ) [71]), and were less likely to know multiple neighbours compared to control participants.

Given the limited sample size, it is not optimal to control for all variables upon which the two groups differ, therefore, the Bayesian Information Criterion (BIC) is used to determine which covariates to include [72]. The BIC, which measures goodness of fit, is estimated for different combinations of baseline variables, while accounting for the number of variables included in the model. A similar method is adopted in [24]. The set of variables which result in the lowest BIC is infant gender, program duration, emotional attachment, number of neighbours known, and exercise.

### Data collection

The survey was piloted between November 2012 and January 2013 with a convenience sample of parents (n = 5), PFL program staff (n = 7), and PFL pilot families (n = 5). Data collection commenced 1^st^ February 2013 and ended 30^th^ November 2013 when the target sample was exhausted. Participants were visited in their homes or a community centre by a researcher who was blind to treatment assignment on two occasions over a three weekday period. On the first day, participants were given diaries and asked to record the next day’s activities. On the third day the survey was completed. Participants were given a €20 voucher as a thank you for their participation. The survey (~50 minutes) consisted of: an adapted Day Reconstruction Method (DRM) [3], mood yesterday questions, global questions of life satisfaction, and the Parenting Stress Index (PSI) [73].

### Instruments

#### Adapted day reconstruction method (DRM) [3]

The DRM was adapted for this study based on the research question, literature review, and piloting. To assist with completion, participants were asked to keep a diary of the study day which they could use during the survey as a prompt to describe each of the day’s episodes in terms of the time it began and ended, the type of activity they were participating in, where they were, and who they were interacting with, either in person or on the phone. Participants were also asked to rate each episode in terms of 12 affect states including 5 positive states (happy, affectionate, competent, relaxed, in control), and 7 negative states (depressed, impatient, criticized, angry, frustrated, irritated, stressed) on a 7-point Likert scale from not at all to very strongly. On average, episodes lasted 80 minutes, and participants recorded approximately 11 episodes per day, which is in line with prior DRM research e.g., [59].

The 12 individual affect states are examined separately across the entire day and are averaged to create positive and negative affect scores. The difference between positive and negative affect is also calculated to provide an overall measure of utility, known as net affect. All scores are weighted by episode length, such that longer episodes contribute more towards a participant’s affect state than shorter episodes.

To overcome the potential issue of different participants interpreting the affect states in a different manner, we also use the U-index to capture the proportion of time a participant spends in an unpleasant state [42]. An episode is categorized as unpleasant if the highest rated affect state is a negative one. Crucially, all participants need not view a certain scale point as being precisely equivalent, they only need to have the same ranking of affect states. The U-Index is also weighted by episode length. For all scores derived from the DRM, we compare the treatment and control groups for the entire day and for subsets of episodes spent with and without the PFL target child.

#### Measures of mood yesterday

To explore the utility of a less intensive proxy of experienced affect, participants were asked to indicate the percentage of time they spent in a bad mood, a little low or irritable, in a mildly pleasant mood, and in a very good mood in relation to the day overall and in terms of the time they spent with their child(ren). The mood variable is a continuous measure ranging from 0–100% indicating the proportion of time spent in a good mood (mildly pleasant mood plus a very good mood).

#### Global life satisfaction

To assess participants’ global evaluations of their well-being, participants were asked to indicate the degree to which they were satisfied with their “life as a whole”, “life at home”, and their “life as a parent” on a 4-point Likert scale from very unsatisfied to very satisfied. Three binary variables (satisfied plus very satisfied versus unsatisfied plus very unsatisfied) are created.

#### Parenting stress index short form (PSI) [73]

The PSI includes 36 items rated on a 5-point Likert scale ranging from strongly disagree to strongly agree. The scale yields a total stress score (α = 0.90) and three subscale scores: Parental Distress (α = 0.90), Parent-Child Dysfunctional Interaction (α = 0.90), and Difficult Child (α = 0.89). Responses are summed to generate scores for each subscale and the Total Stress score. A binary variable is created to represent mothers scoring above a cut-off of 90, indicating a high level of stress. The PSI also contains a measure of defensive responding [73] derived from the widely used Crowne-Marlowe Social Desirability Scale. These questions pertain to routine parenting experiences, a denial of which can be interpreted as defensive rather than accurate responding. A score of 10 or below on this scale indicates defensive responding.

S2 Table presents the correlations between the various well-being measures and finds a strong correlation among the measures derived from the DRM. The DRM measures are moderately correlated with the measure of mood yesterday, yet only weakly correlated with the global measure of life satisfaction and the PSI measures. These correlations suggest that the global and experienced measures of well-being may represent different measures.

## Data analytic Procedures

### Empirical approach

This study adopts an intention-to-treat approach. The standard treatment effect framework describes the observed outcome Y_i_ of participant i ∈ I by:

$${\text{Y}}_{\text{i}}={\text{D}}_{\text{i}}{\text{Y}}_{\text{i}}\left(1\right)+\left(1-{\text{D}}_{\text{i}}\right){\text{Y}}_{\text{i}}\left(0\right)\;\text{i}\in \text{I}=\left\{1\ldots \text{N}\right\}$$ (1)

where I = {1 … N} denotes the sample space, D_i_ denotes the treatment assignment for participant i (D_i_ = 1 for the intention-to-treat sample, D_i_ = 0 otherwise) and (Y_i_(0), Y_i_(1)) are potential outcomes for participant i. We test the null hypothesis of no treatment effect on maternal well-being via:

$${\text{Y}}_{\text{i}}=\beta_{0}+\beta_{1}{\text{D}}_{\text{i}}+\epsilon_{\text{i}}$$ (2)

Eq 2 is estimated using t-tests/OLS regressions for continuous outcomes and chi-squared tests/logistic regressions for binary outcomes, both excluding and including relevant group differences. Permutation-based hypothesis testing is also used as it does not depend on distributional assumptions and thus facilitates the estimation of treatment effects in small samples [74]. A permutation test relies on the assumption of exchangeability under the null hypothesis. Permutation tests work by calculating the observed test statistic which compares the outcomes of the treatment and control group. Then, the data are repeatedly shuffled so that the treatment assignment of some participants is switched between the groups. The p-value for the permutation test is the proportion of permutations that have a test statistic more extreme than the observed test statistic in the original sample. Permutation tests based on 100,000 replications are computed.

The permutation procedure relies on the exchangeability properties of the joint distribution of outcomes and treatment assignment. When the exchangeability property is not obvious, e.g. the two groups differ on certain characteristics, a conditional inference that relies on restricted classes of permutations can be implemented. This procedure uses the conditional exchangeability property and tests for program effects while controlling for variables upon which the joint distribution of outcomes and treatment assignment is exchangeable. Conditional permutation testing first partitions the sample into subsets, termed orbits, each consisting of participants with common background measures. Under the null hypothesis of no treatment effect, treatment and control outcomes have the same distributions within an orbit. Thus, the exchangeability assumption is restricted to strata defined by the controls. In our conditional analysis we include the six control variables identified using the BIC procedure. One binary variable is used to produce the orbits: child gender. However, using orbits proves problematic with multiple conditioning variables as the strata become too small leading to a lack of variation within each orbit. To circumvent this problem we assume a linear relationship between the remaining five conditioning variables and the outcomes. The control set includes program duration, emotional attachment score, number of neighbours known, and exercise. Thus, we partition the data into orbits on the basis of the child’s gender and then regress each outcome on the five variables assumed to share a linear relationship with the outcomes. Next, the residuals are permuted, based on 100,000 replications, from this regression within the orbits. This method is referred to as the Freedman–Lane procedure [75] and was found to be statistically sound in a series of Monte Carlo studies [76]. The results below include both conditional and unconditional permutation testing.

### Additional analysis

Analysing the impact of the program on multiple well-being measures increases the likelihood of a Type-1 error and studies of RCTs have been criticized for overstating treatment effects due to this ‘multiplicity’ effect [77]. To address this issue we employ the stepdown procedure [78] whereby we calculate a t-statistic for each null hypothesis in a family of outcomes and placing them in descending order. The outcome measures included in each family should be correlated and measure a similar construct. Thus, the well-being measures are placed into 14 stepdown families and the procedure is conducted only on the families where significant differences are identified in the individual tests. Using the permutation testing method, the largest observed t-statistic is compared with the distribution of maxima permuted t-statistics. If the probability of observing this statistic by chance is high (p ≥ 0.1), we fail to reject the joint null hypothesis that the treatment has no impact on any outcome in the family of measures being tested. If the probability of observing this t-statistic is low (p < 0.1), we reject the joint null hypothesis and proceed by excluding the most significant individual hypothesis and test the subset of hypotheses that remain for joint significance. This process of dropping the most significant individual hypothesis continues until only one hypothesis remains. ‘Stepping down’ through the hypotheses allows us to isolate the hypotheses that lead to a rejection of the null. This method is superior to Bonferroni adjustment as it accounts for interdependence across outcomes.

In addition to examining differences in well-being, we also explore patterns of time use across the treatment and control groups regarding interactions (with the PFL target child, the participant’s partner, and other family members), locations (home and workplace), and activities (looking after and playing with children, relaxing/socializing, housework/cooking, exercising and commuting).

We apply two-tailed tests for all analyses as we are not proposing a specific directional hypothesis regarding the program’s impact on well-being.

## Results

### Descriptive statistics on affect measures

**Fig 2:**
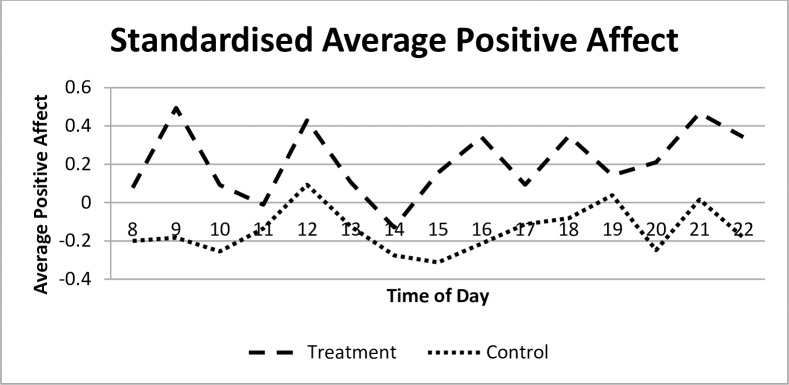
Standardized average positive affect for treatment and control groups.

For each episode, participants report a score for a range of affect states which are classified as positive or negative. To generate descriptive statistics, the positive and negative affect values are standardized for the entire sample to have a zero mean and a standard deviation of one. Every episode recorded is assigned an hour corresponding to the midpoint of the episode. For each midpoint hour from 08:00 to 22:00, the average positive and negative affect is calculated separately for the treatment and control groups. Fig 2 illustrates the pattern of average positive affect over the course of the study day and shows that the treatment group report higher positive affect scores at every hour, compared to the control group.

**Fig 3:**
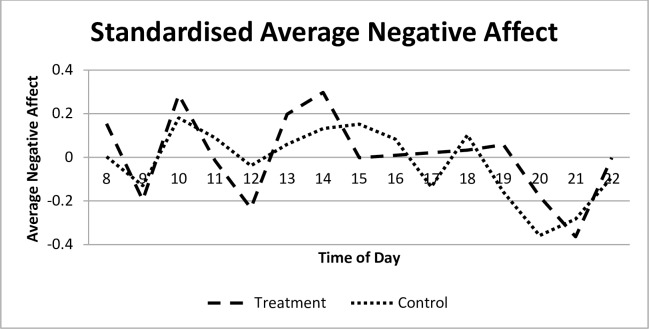
Standardized average negative affect for treatment and control groups.

Conversely, Fig 3 indicates that there is no clear difference in negative affect between the two groups. Both the treatment and control groups display a similar pattern of mid-morning and mid-afternoon peaks in negative affect, followed by an evening decline as is typical (e.g. [59; 79]).

### Estimation of treatment effects

Tables 1–4 present estimates of treatment effects for experienced and global measures of maternal well-being. The unconditional means and standard deviations are reported throughout. Four columns of p-values are presented in each table representing the statistical significance of the treatment effect from an unconditional t-test/chi-squared test, an unconditional permutation test, a conditional t-test/chi-squared test, and a conditional permutation test, respectively. Given the few observed differences between the treatment and control groups at baseline, the conditional results represent the most reliable set of findings. Overall, the t-tests and the permutation tests produce very similar results.

**Table 1:** Treatment Effects for Experienced Well-being: Mood Yesterday, Net Affect and U-Index.

	*M*_TREATMENT_ (*SD*)	*M*_CONTROL_ (*SD*)	*p*^*a*^	*p*^*b*^	*p*^*a*^	*p*^*b*^
			Unconditional		Conditional	
*Mood Yesterday*						
Portion of day spent in a positive mood	0.76 (0.18)	0.71 (0.25)	0.321	0.308	0.047**	0.035**
Portion of day spent with children in a positive mood	0.83 (0.21)	0.84 (0.19)	0.821	0.827	0.783	0.673
*Net Affect*						
Net Affect	3.03 (1.41)	2.84 (1.37)	0.511	0.512	0.329	0.269
Net affect during time spent with *PFL* child	2.98 (1.58)	2.95 (1.38)	0.916	0.917	0.603	0.637
Net affect during time spent without *PFL* child	3.00 (1.78)	2.68 (1.59)	0.353	0.356	0.355	0.188
*U-Index*						
U-Index	0.10 (0.14)	0.09 (0.18)	0.686	0.689	0.777	0.315
U-Index during time spent with *PFL* child	0.10 (0.16)	0.08 (0.18)	0.453	0.461	0.703	0.758
U-Index during time spent without *PFL* child	0.11 (0.24)	0.12 (0.27)	0.874	0.875	0.907	0.235

*Notes*: The sample size is 101 (Treatment = 46, Control = 55), except when we restrict the analysis to time spend without the *PFL* child as 5 control participants did not record any episodes without their *PFL* child, therefore n = 96 (Treatment = 46, Control = 50), and apart from Mood Yesterday (Treatment = 45, Control = 55). ‘M’ indicates the unconditional mean. ‘SD’ indicates the unconditional standard deviation.

^a^ two-tailed t-test p-value.

^b^ two-tailed p-value from an individual permutation test with 100,000 replications.

*** Significant at the 1 percent level.

** Significant at the 5 percent level.

* Significant at the 10 percent level.

**Table 2:** Treatment Effects for Experienced Well-being: Positive Affect.

	*M*_TREATMENT_ (*SD*)	*M*_CONTROL_ (*SD*)	*p*^*a*^	*p*^*b*^	*p*^*a*^	*p*^*b*^
			Unconditional		Conditional	
*Overall*						
Positive affect	3.94 (0.96)	3.66 (0.95)	0.151	0.150	0.214	0.188
Positive affect during time spent with *PFL* Child	3.97 (1.02)	3.77 (1.00)	0.336	0.336	0.373	0.414
Positive affect during time spent without *PFL* child	3.84 (1.13)	3.48 (0.92)	0.088*	0.090*	0.184	0.122
*Positive affect states*						
Happy	4.03 (1.00)	3.59 (1.12)	0.043**	0.041**	0.064*	0.044**
Affectionate	3.75 (1.49)	3.43 (1.38)	0.271	0.273	0.530	0.430
Competent	4.40 (1.04)	4.18 (1.12)	0.324	0.320	0.402	0.408
In Control	4.25 (1.16)	4.04 (1.19)	0.379	0.378	0.432	0.444
Relaxed	3.24 (1.16)	3.04 (1.16)	0.410	0.409	0.322	0.302
*Positive affect states during time spent with PFL child*						
Happy	3.99 (1.22)	3.59 (1.17)	0.094*	0.096*	0.091*	0.108
Affectionate	4.25 (1.42)	3.98 (1.40)	0.340	0.341	0.562	0.588
Competent	4.34 (1.09)	4.13 (1.22)	0.358	0.353	0.393	0.412
In Control	4.25 (1.20)	4.13 (1.17)	0.607	0.607	0.756	0.761
Relaxed	2.94 (1.34)	3.00 (1.21)	0.834	0.836	0.910	0.960
*Positive affect states during time spent without PFL child*						
Happy	3.98 (1.07)	3.50 (1.25)	0.045**	0.045**	0.074*	0.038*
Affectionate	3.08 (1.89)	2.57 (1.59)	0.159	0.162	0.450	0.293
Competent	4.31 (1.40)	4.16 (1.15)	0.550	0.553	0.675	0.640
In Control	4.17 (1.44)	4.00 (1.29)	0.522	0.522	0.599	0.547
Relaxed	3.67 (1.59)	3.18 (1.27)	0.100	0.103	0.120	0.094*

*Notes*: The sample size is 101 (Treatment = 46, Control = 55), except when we restrict the analysis to time spend without the *PFL* child as 5 control participants did not record any episodes without their *PFL* child, therefore n = 96 (Treatment = 46, Control = 50). ‘M’ indicates the unconditional mean. ‘SD’ indicates the unconditional standard deviation.

^a^ two-tailed t-test p-value.

^b^ two-tailed p-value from an individual permutation test with 100,000 replications.

*** Significant at the 1 percent level.

** Significant at the 5 percent level.

* Significant at the 10 percent level.

**Table 3:** Treatment Effects for Experienced Well-being: Negative Affect.

	*M*_TREATMENT_ (*SD*)	*M*_CONTROL_ (*SD*)	*p*^*a*^	*p*^*b*^	*p*^*a*^	*p*^*b*^
			Unconditional		Conditional	
*Overall*						
Negative affect	0.91 (0.79)	0.82 (0.76)	0.547	0.551	0.852	0.894
Negative affect during time spent with *PFL* child	0.98 (0.88)	0.82 (0.73)	0.309	0.323	0.852	0.658
Negative affect during time spent without *PFL* child	0.84 (0.97)	0.80 (0.92)	0.831	0.833	0.857	0.671
*Negative affect states*						
Stressed	1.47 (1.25)	1.24 (1.08)	0.320	0.329	0.932	0.864
Irritated	1.29 (1.12)	1.08 (1.05)	0.338	0.343	0.734	0.805
Frustrated	1.26 (1.02)	1.10 (1.00)	0.422	0.426	0.866	0.812
Angry	0.66 (0.84)	0.55 (0.84)	0.504	0.510	0.826	0.972
Impatient	1.27 (1.15)	1.32 (1.02)	0.829	0.830	0.590	0.583
Depressed	0.23 (0.37)	0.28 (0.50)	0.627	0.622	0.177	0.196
Criticized	0.18 (0.40)	0.16 (0.36)	0.781	0.786	0.444	0.526
*Negative affect states during time spent with PFL child*						
Stressed	1.61 (1.45)	1.25 (1.08)	0.155	0.167	0.570	0.438
Irritated	1.36 (1.22)	1.04 (0.98)	0.153	0.164	0.293	0.311
Frustrated	1.37 (1.19)	1.11 (1.00)	0.233	0.245	0.601	0.447
Angry	0.66 (0.87)	0.56 (0.85)	0.584	0.593	0.717	0.987
Impatient	1.43 (1.26)	1.36 (1.09)	0.783	0.787	0.854	0.910
Depressed	0.24 (0.53)	0.24 (0.49)	0.989	0.990	0.229	0.421
Criticised	0.22 (0.49)	0.17 (0.39)	0.600	0.611	0.529	0.712
*Negative affect states during time spent without PFL child*						
Stressed	1.36 (1.61)	1.23 (1.31)	0.672	0.674	0.746	0.644
Irritated	1.16 (1.38)	1.03 (1.33)	0.634	0.636	0.977	0.836
Frustrated	1.10 (1.31)	1.07 (1.29)	0.895	0.896	0.827	0.671
Angry	0.70 (1.21)	0.58 (1.15)	0.620	0.625	0.945	0.993
Impatient	1.15 (1.46)	1.12 (1.29)	0.932	0.934	0.801	0.895
Depressed	0.26 (0.57)	0.44 (0.91)	0.255	0.256	0.244	0.176
Criticised	0.14 (0.58)	0.13 (0.34)	0.922	0.929	0.871	0.728

*Notes*: The sample size is 101 (Treatment = 46, Control = 55), except when we the restrict analysis to time spend without the *PFL* child as 5 control participants did not record any episodes without their *PFL* child, therefore n = 96 (Treatment = 46, Control = 50). ‘M’ indicates the unconditional mean. ‘SD’ indicates the unconditional standard deviation.

^a^ two-tailed t-test p-value

^b^ two-tailed p-value from an individual permutation test with 100,000 replications.

*** Significant at the 1 percent level.

** Significant at the 5 percent level.

* Significant at the 10 percent level.

**Table 4:** Treatment Effects for Global Well-being: Life Satisfaction and Parenting Stress Index.

	N (*n*_TREATMENT_*/ n*_CONTROL)_	*M*_TREATMENT_ (*SD*)	*M*_CONTROL_ (*SD*)	*p*^*a*^		*p*^*b*^	*p*^*a*^	*p*^*b*^
				Unconditional			Conditional	
*Life Satisfaction*								
Satisfaction with Life as a Parent	100 (45/55)	0.98 (0.15)	0.89 (0.31)	0.126	0.118		0.190	0.160
Satisfaction with Home Life	100 (45/55)	0.96 (0.21)	0.89 (0.31)	0.251	0.234		0.303	0.319
Satisfaction with Life Overall	100 (45/55)	0.93 (0.25)	0.89 (0.31)	0.465	0.477		0.650	0.704
*PSI subdomains*								
Parent-Child Dysfunctional Interactions	99 (45/54)	18.04 (5.44)	17.22 (5.40)	0.402	0.456		0.855	0.735
Difficult Child	94 (43/51)	22.42 (8.34)	22.18 (7.03)	0.944	0.881		0.605	0.697
Parental Distress	100 (45/55)	24.82 (8.39)	24.67 (8.50)	0.907	0.932		0.661	0.548
Total Parental Stress	93 (42/51)	64.52 (18.17)	64.02 (17.95)	0.888	0.894		0.641	0.646
Stress Cut-off	93 (42/51)	0.10 (0.30)	0.08 (0.27)	0.752	0.827		0.601	0.900
Defensive Responding	93 (42/51)	14.76 (5.24)	14.64 (5.05)	0.967	0.972		0.621	0.518
Defensive Responding Cut-off	93 (42/51)	0.24 (0.43)	0.27 (0.45)	0.731	0.694		0.980	0.945

*Notes*: **‘**N’ indicates the sample size. ‘M’ indicates the unconditional mean. ‘SD’ indicates the unconditional standard deviation.

^a^ two-tailed t-test p-value

^b^ two-tailed p-value from an individual permutation test with 100,000 replications.

*** Significant at the 1 percent level.

** Significant at the 5 percent level.

* Significant at the 10 percent level.

Table 1 compares the treatment and control groups in terms of their mood yesterday, net affect, and U-Index for the day as a whole and also for time spent with and without the PFL child. Both groups report spending approximately three-quarters of the study day in a positive mood. This increases to four-fifths during episodes spent with children. The treatment group reports spending a significantly higher proportion of their day in a positive mood, relative to the control group, yet this difference is only significant in the conditional models.

In terms of the DRM measures, on average, participants in both groups report a net affect score of approximately 3 which implies that participants experience positive emotions three points more intensively on the 0–6 Likert scale than negative emotions. Both groups spend approximately 10% of their day in an episode where the strongest emotion is a negative one, as shown by the U-Index. Both groups experience a slight decline in net affect and a corresponding slight rise in the U-Index in episodes when they are without their PFL child. No significant treatment effects are identified for the net affect or U-Index measures.

Table 2 compares the treatment and control groups in terms of their overall positive affect and individual positive affect states for the day as a whole and also time spent with and without the PFL child. Feelings of competence and control receive the highest ratings, while feeling relaxed receives the lowest. This pattern differs depending on whether participants were with/without their PFL child, with participants reporting substantially higher levels of affection during episodes with the PFL child. A treatment effect is identified in the unconditional models for overall positive affect for episodes spent without the PFL child. In the conditional models, the p-values are slightly larger and not statistical significant at conventional levels.

For individual positive affect states, we find that treatment participants report significantly higher levels of happiness for the day overall and during times spent without the PFL child in the unconditional and conditional models. In all models, apart from the conditional permutation model, the treatment group also report higher levels of happiness during times spent with the PFL child. In the conditional permutation model, the treatment group are significantly more relaxed during episodes without their child. The groups do not significantly differ on the remaining positive affect states.

Tests comparing positive affect states when with and without the PFL target child (not reported) show that participants from both groups are significantly less affectionate during episodes without their PFL child, yet the control group experience a larger decline. Additionally, control group participants feel significantly less in control when they are without their PFL child, while treatment participants are significantly more relaxed when without their PFL child.

Table 3 compares the treatment and control groups in terms of their negative affect and individual negative affect states for the entire day and for time spent with and without their PFL child. No significant treatment effects are identified. Both treatment and control participants tend to give the highest ratings to feeling stressed and impatient, with depressed and criticised receiving the lowest ratings. Overall, ratings of negative affect states seem to be slightly less intense when participants were not with their PFL child, although none of these differences are significant for either group (not reported).

Table 4 presents the results for the global measures of life satisfaction and the standardized measure of parenting stress. In terms of life satisfaction, the majority of participants in both groups report that they are satisfied with their life overall, as a parent, and at home. A slightly higher proportion of treatment participants than control participants report that they are satisfied across all three categories, however none of these differences are statistically significant. Note that only 9 participants across both groups report being either unsatisfied or very unsatisfied with their life overall, thus the small cell size should be noted when interpreting the results. In addition, when ordered logit models are calculated using the original 4-point scale, there is a statistical significance difference between the treatment and control group regarding satisfaction with life as a parent in the unconditional and conditional models.

The treatment and control groups report comparable levels of parenting stress (PSI), and approximately 10% report clinically significant levels. There are no significant treatment effects for any of the PSI scores. In addition, 24% of the treatment group and 27% of the control group meet the cut off for defensive responding suggesting that these participants may be positively biasing their responses based on their perception of socially desirable parenting experiences. Importantly, however, there are no significant differences between the groups in terms of defensive responding, suggesting no evidence of systematic mis-reporting.

### Additional analysis

#### Stepdown analysis

Table 5 presents the unconditional and conditional stepdown results for the measures upon which we identified significant differences in the individual tests. The first p-value in the conditional mood yesterday stepdown family is significant following adjustment for multiple comparisons, and is driven by the significant finding for the portion of day spent in a positive mood. In contrast, the stepdown families for positive affect states for the day as a whole or for episodes with and without their PFL child are not significant when the unconditional and conditional stepdown procedure is applied.

**Table 5:** Stepdown Results.

	Stepdown Test *p*^*a*^	Stepdown Test *p*^*b*^
*Mood Yesterday*		
Portion of Day Spent in a Positive Mood	~	0.066*
*Positive affect states*		
Happy	0.138	0.146
*Positive affect states during time spent with PFL child*		
Happy	0.294	~
*Positive affect states during time spent without PFL child*		
Happy	0.162	0.133
Relaxed	~	0.279

Notes

^a^ two-tailed p-value from an unconditional stepdown permutation test with 100,000 replications.

^b^ two-tailed p-value from a conditional stepdown permutation test with 100,000 replications.

*** Significant at the 1 percent level.

** Significant at the 5 percent level.

* Significant at the 10 percent level.

#### Time use

The few observed treatment effects may be driven by differences in time use across the two groups. Yet, as shown in Table 6, the treatment group spend approximately the same proportion of episodes with their PFL child (62%) as do the control group (66%). In addition, there are no differences regarding the proportion of episodes spent caring for or playing with their children. The conditional results show that the treatment group are significantly more likely to spend an episode with their relatives and a higher proportion of their episodes in work, yet less than 6% of all episodes are spent at work. There are also no differences in terms of daily activities (relaxing/socializing, housework/cooking, commuting, exercising).

**Table 6:** Time Use Amongst Treatment and Control Groups.

	*%*_TREATMENT_	*%*_CONTROL_	*Unconditional p*^*a*^	*Conditional p*^*b*^
*Interaction*				
With *PFL* child	61.89	66.28	0.125	0.214
With partner	16.70	22.09	0.019**	0.244
With relatives	22.99	16.45	0.008***	0.026**
Alone	9.49	10.89	0.445	0.217
*Location*				
At home	66.60	64.95	0.564	0.554
At work	5.89	3.16	0.029**	0.045**
*Activities*				
Looking after children	44.20	46.84	0.399	0.369
Playing with children	8.84	8.97	0.962	0.658
Relaxing/socializing	24.95	25.42	0.881	0.927
Housework/cooking	26.92	29.40	0.376	0.685
Commuting	12.77	13.95	0.540	0.598
Exercising	1.57	2.16	0.501	0.370

*Notes*: Unconditional percentages are reported.

^a^ two-tailed p-value from an individual unconditional permutation test with 100,000 replications.

^b^ two-tailed p-value from an individual conditional permutation test with 100,000 replications.

*** Significant at the 1 percent level.

** Significant at the 5 percent level.

* Significant at the 10 percent level.

## Discussion

It has been proposed that aggregated measures of experienced affect can be utilized as a measure of policy effectiveness [3] and that such measures replace traditional quality of life questions in health care evaluations [80]. Yet, to date, no study has attempted to integrate these insights into a formal policy evaluation. This paper examines the utility effects of a targeted early intervention program using multiple measures of well-being. In sum, we find limited evidence that the PFL intervention affects global measures of maternal well-being. However, the intervention does generate higher levels of experienced positive affect using a Day Reconstruction Method. Specifically, participants in the treatment group experience higher levels of happiness for the day overall and when they are with and without the PFL child. Participants also report feeling more relaxed during episodes without the PFL child, yet these results do not survive the stepdown procedure. These results are consistent with the findings for positive mood yesterday, where we observe significant treatment effects in the individual and stepdown results, yet not during times spent with children. There are no treatment effects for negative aspects of well-being irrespective of the measure used. Lastly, although higher proportions of the treatment group report being satisfied with their lives, these differences did not reach significance.

The lack of treatment effects on negative measures of well-being is broadly in keeping with the HVP literature. Systematic reviews have found that home visiting is typically not effective in ameliorating negative emotional states [29, 35]. Thus our findings are consistent with the view that targeted and intensive therapeutic supplements are needed in order for HVPs to alleviate negative states such as depression [35]. Notwithstanding this, our findings demonstrate that a HVP may have an impact on some dimensions of positive affect, which questions the prevailing assumption, based predominantly on deficit measures of well-being, that HVPs do not influence parents’ emotional states [39].

While there are no differences in the amount of time participants spend with their children in either group, the results suggest that the higher positive affect experienced by the treatment group may be driven by differences in the quality of the episodes rather than the quantity of episodes. Indeed the intervention aims to improve the quality and type of parent-child interactions rather than the amount of time spent with the child. For example, many of the Tip Sheets discuss the importance of reading to your child, talking to your child, and creating a secure base. It is also possible that gains to maternal well-being, and happiness in particular, are accrued indirectly, via the program’s identified impact on the children’s cognitive, emotional, and physical health [5, 6]. However, directionality may be obscured due to the dynamic and bidirectional interplay between child and maternal well-being [81].

The PFL intervention aims to heighten parents’ awareness of being actively engaged when interacting with their child. If such investment confers an increased effort on the parents, treatment mothers may particularly value times when they are not actively being a parent. This lends some supports to the finding that the treatment group feel more relaxed than the control group when without the PFL child. It is also possible that, through Tip Sheets and mentor support, the mothers are encouraged to use their non-parenting time for self-care, relaxation, and social relationships. These supports may result in positive emotional experiences as rich social relationships are integral to optimizing happiness [13], and socializing and relaxing typically receive the highest ratings of experienced positive affect on the DRM [3]. While there are no differences in time use between the two groups, it is possible that the quality of these non-parenting experiences differ in some unobserved way.

Another key question concerns the intervention’s effect on experienced positive affect and assessments of yesterday’s mood, but not the global assessments of well-being such as life satisfaction. It is possible that the DRM provides a more sensitive measure of well-being which avoids the cognitive biases that impinge upon global assessments of life satisfaction. Such biases may operate less intensively on measures of yesterday’s mood [62]. Another hypothesis is that global and experienced well-being are independent constructs, as reflected in the recent conceptual shift which recognizes experienced well-being and global well-being as distinct psychological phenomena [61]. Applied to our study, the absence of treatment effects for global well-being may be counterintuitive, if we believe that the life satisfaction question should have encouraged participants to focus on their participation in the program, its association with greater parenting competency, and anticipation of future benefits. Indeed, one study has found that while spending time with children was not highly pleasurable, it was thought of as rewarding [82]. Thus, the authors postulate that parenting may have a more positive influence on global aspects of well-being by providing individuals with a sense of purpose, connection, and contribution to personal goals. Interestingly, one other study has found that the cost of parenthood—in this case monetary—appears to motivate parents to idealize global judgements of how rewarding parenting is [83]. It is also possible that participants habituate quickly to their circumstances [84]—in this case treatment status—and thus the effects on global well-being may have dissipated over time as, on average, the participants have spent four years in the program.

Given the absence of experimental studies examining the causal impact of policy interventions on experienced well-being, it is difficult to give precise comparisons to the magnitude of our results. Comparing our happiness effect (0.42 points more than average well-being) to the well-being effects observed in the original non-experimental DRM study [3], we identify a similar magnitude to the effect of commuting (.49 points less than average well-being) and being alone (.48 points less than average). In addition, the treatment participants’ average levels of happiness for times when they are without the study child (3.98), are very similar to those reported in the original DRM sample of employed women (3.96) [79]. This suggests that the treatment may raise the levels of well-being of a disadvantaged group closer to those that are typical of the population.

While this study is the first to our knowledge to test for the causal impact of a policy intervention on multiple measures of well-being, some methodological issues should be acknowledged. The study relies on self-report measures which may be contaminated by social desirability bias when participants are not blinded to their treatment status. However, we demonstrate that there are no systematic differences in social desirability between the treatment and control groups according to the defensive responding validity measure embedded within the PSI. An additional issue which is common in many trials is small sample size. This issue is a particular concern in the present study as the sample in the sub-study is smaller than in the original PFL trial. Yet we find that few individual characteristics predict selection into the sub-study, and the randomization assumption of baseline equivalence still holds in the reduced sample. In addition, the sample size is equivalent to seminal studies of other early intervention programs, such as the Perry Preschool program and the Abecedarian program. A discussion on the use of small samples in experimental trials may be found in [10] and [24]. The permutation testing method also helps to address this issue. A further concern is the risk of overstating the program’s impact due to multiple hypothesis testing. We address this issue using the stepdown procedure and highlight the significance of failing to account for this issue. The stepdown analysis shows that only the result for mood yesterday remains significant after adjustment.

If the identified treatment effect for experienced positive mood is valid, this may confer meaningful benefits for mothers. Evidence suggests that positive emotions create an upward positive spiral in emotional well-being by enhancing an individual’s cognitive coping strategies [85]. Over time a causal relationship may develop between positive affect and behaviors linked to successful outcomes such as higher quality relationships, superior income and productivity, greater community participation, and improved health and mortality [86, 87]. Thus, the treatment effect identified here may have important implications for the cost-benefit analysis of the PFL program and similar HVPs in the future. A full cost-benefit analysis of the program will be conducted when the final outcome data are available. Note that the majority of cost savings (if realised) are likely to be derived from improvements in child outcomes than improvements in parental well-being.

Using RCTs to examine the well-being effects of policy interventions is a growing area. Our findings demonstrate the importance of measurement and conceptualization of well-being and of inferential techniques. Further research is needed to reconcile differences on global versus experienced measures of well-being and on positive and negative affect. These issues are important across many domains, including labor market and health interventions, where there is also likely to be a psychic benefit of successful program outcomes on top of the core measures being targeted.

## Supporting Information

S1 Data (XLS)

S1 Protocol (DOC)

S2 Protocol (DOC)

S1 Table (DOCX)

S2 Table (DOCX)
