# Supplementary material for: Can Early Intervention Improve Maternal Well-Being? Evidence from a Randomized Controlled Trial
Source: PLoS One. 2017 Jan 17;12(1):e0169829. doi: 10.1371/journal.pone.0169829 (PMC5241149; doi:10.1371/journal.pone.0169829)
Supplement: S2 Protocol — (DOC) [file pone.0169829.s003.doc]

**UCD Geary Institute**

**Institiúid Geary UCD**

UCD Geary Institute,

University College Dublin,

Belfield, Dublin 4, Ireland

T +353 1 716 4637

F +353 1 716 1108

E-mail: geary@ucd.ie

Institiúid Geary UCD,

An Coláiste Ollscoile, Baile Átha Cliath,

Belfield, Baile Átha Cliath 4, Eire

www.ucd.ie/geary

**S2 Protocol**

**TRIAL PROTOCOL**


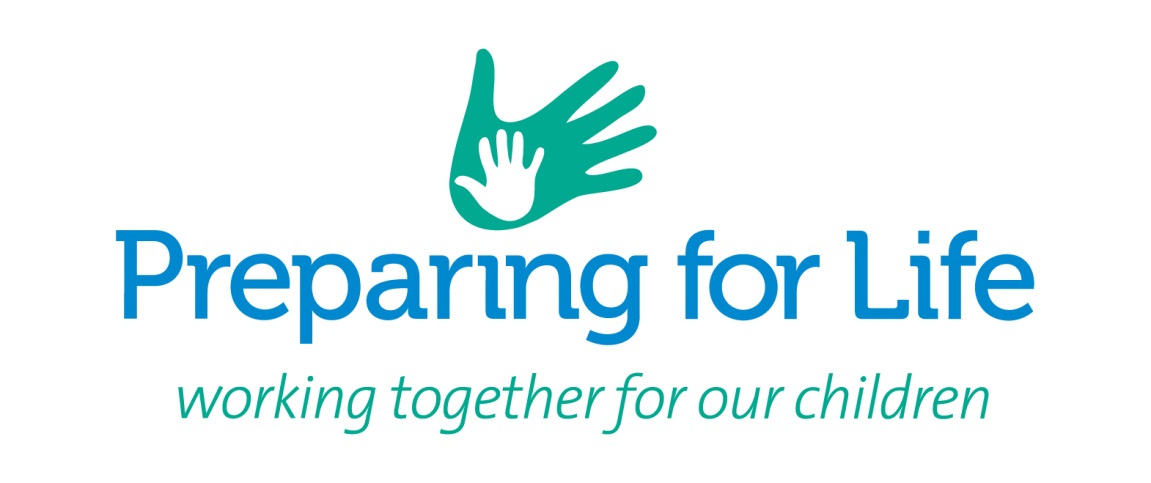


**Study Title:** Evaluation of the Preparing for Life early childhood intervention programme

**Sponsor:** UCD Geary Institute

**Date:** 31 July 2007


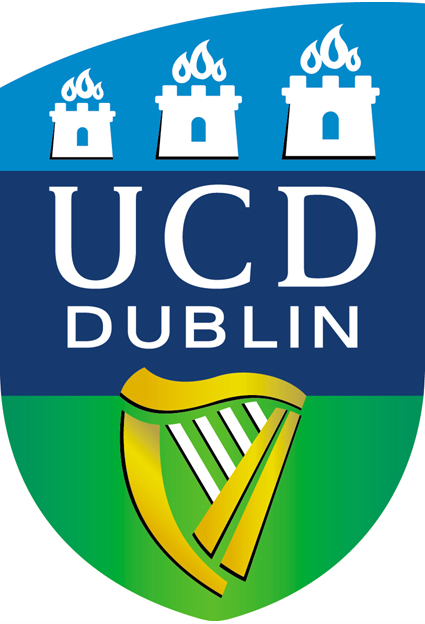


CONTACT LIST

**Principal Investigator**

Dr Orla Doyle

UCD Geary Institute

University College Dublin

Dublin 4

Ireland

Tel: +353 17164637

Email: [orla.doyle@ucd.ie](mailto:orla.doyle@ucd.ie)

**Sponsor**

| UCD Geary Institute  University College Dublin  Dublin 4  Ireland  Contact person: Colm Harmon  Tel: +353 17164625  Email: [colm.harmon@ucd.ie](mailto:colm.harmon@ucd.ie) |
| --- |

**Funder**

| Northside Partnership  Coolock Development Centre  Bunratty Drive  Coolock  Dublin 17  Co. Dublin  Tel: 0035318485661  Email: [info@nspartnership.ie](mailto:info@nspartnership.ie) |
| --- |

**Other Investigators**

Professor Richard Tremblay

UCD School of Public Health and Population Science

University College Dublin

Dublin 4

Ireland

Email: [richard.tremblay@ucd.ie](mailto:richard.tremblay@ucd.ie)

Prof. Sylvana Cote

Département de Médecine Sociale e Préventive

Univeristy of Montreal

3050 Boul. Edouard-Montpetit

Montreal

Quebec,

Canada

Email: [sylvana.cote1@umontreal.ca](mailto:sylvana.cote1@umontreal.ca)

Prof. Colm Harmon

UCD Geary Institute

University College Dublin

Belfield

Dublin 4

Ireland

Email: [colm.harmon@ucd.ie](mailto:colm.harmon@ucd.ie)

Prof. Cecily Kelleher

UCD School of Public Health and Population Science

University College Dublin

Belfield

Dublin 4

Ireland

Email: [Cecily.Kelleher@ucd.ie](mailto:Cecily.Kelleher@ucd.ie)

Prof. James Heckman

Department of Economics,

University of Chicago

1126 East 59^th^ Street

Chicago, Illinois

USA

Email: [jjheckman@uchicago.edu](mailto:jjheckman@uchicago.edu)

Dr. Hannah Lambert

UCD Geary Institute

University College Dublin

Belfield

Dublin 4

Ireland

Email: [Hannah.lambert@ucd.ie](mailto:Hannah.lambert@ucd.ie)

# 1. TRIAL SUMMARY

| **TITLE** | A randomised trial to determine the effectiveness of the Preparing For Life programme which aims to improve the school readiness skills of socioeconomically disadvantaged children in Dublin, Ireland |
| --- | --- |
| **OBJECTIVES** | **Primary objective**  To test the effectiveness of the Preparing for Life (PFL) programme at improving the school readiness skills and life outcomes of socioeconomically disadvantaged children.  **Secondary objective**  To test the effectiveness of the Preparing for Life (PFL) programme at improving parental skills and the quality of the home environment. |
| **DESIGN** | Randomised controlled trial |
| **SAMPLE SIZE** | 300 (100 in randomised high treatment group; 100 in randomised low treatment group and 100 in external comparison group) |
| **STUDY POPULATION** | Pregnant women residing in the PFL catchment area |
| **ELIGIBILITY CRITERIA** | **Inclusion criteria**   1. Women (age 16+) pregnant between 2008 and 2010 2. Residing in the PFL catchment area 3. Willing to be assigned to either of the study intervention groups 4. Written informed parental/carer consent   **Exclusion criteria**   1. Under the age of 16 2. Not pregnant 3. Not residing in the PFL catchment area 4. No written consent provided. |
| **TREATMENT** | **5 year home visiting mentoring programme & group Triple P parenting programme** |
| **PRIMARY ENDPOINT** | **School Readiness Skills** 1. Cognitive development: measured using Ages and Stages Questionnaire (at 6, 12, 18, 24, 36, 48 months); Developmental Profile 3 (at 12, 18, 24, 36, 48); British Ability Scales (at 48 months); Executive functioning & delay of gratification (48 months); Early Development Instrument- Short Form (at school entry). 2. Physical health and motor skills: measured using Ages and Stages Questionnaire (at 6, 12, 18, 24, 36, 48 months); hospital records (at 48 months); parent-reported child health (at 6, 12, 18, 24, 36, 48); Early Development Instrument- Short Form (at school entry). 3. Socio-emotional development: measured using Temperament and Atypical Behaviour Scale (at 12 months); Difficult temperament (at 6 & 12 months); ASQ-Socio-Emotional scale (at 6, 12, 18, 24, 36, 48 months); Brief Infant Toddler Social and Emotional Assessment (at 12, 18 24, 36 months); Early Development Instrument- Short Form (at school entry). 4. Behavioural skills: measured using Temperament and Atypical Behaviour Scale (at 12 months); Child Behaviour Checklist (at 18, 36, 48 months); Peer Problems and Prosocial Behaviour (at 48 months); Early Development Instrument- Short Form (at school entry). 5. Language development and emergent literacy: measured using Mac-Arthur Bates Communicative Development Inventories (at 12, 18, 24 months); Ages and Stages Questionnaire (at 6, 12, 18, 24, 36, 48 months); British Ability Scales (at 48 months); Early Development Instrument- Short Form (at school entry). |
| **SECONDARY ENDPOINTS** | 1. Birth outcomes: maternity hospital records at birth (birth weight, gestational age, prematurity, Apgar score). 2. Labour outcomes: maternity hospital records at birth (instrumental delivery, caesarean section- elective, emergency). 3. Parenting skills: Adult Adolescence Parenting Inventory (at baseline &12 months); Knowledge of Infant Development (at baseline & 12 months); Parental locus of control (at 6 months); Condon Maternal Attachment Scale (at 6 & 24 months); Parenting Stress Index (at 6, 24 & 48 months); Parenting Daily Hassles Scale (at 18, 36 & 48 months).; Maternal Separation Anxiety (at 18 months); Parental Cognitions and Conduct Towards Infant Scale (at 6 & 24 months); Parenting Styles and Dimensions Questionnaire (at 36 & 48 months); Parental Acceptance -Rejection Questionnaire (at 36 months).  4. Quality of the home environment: Home Observation for Measurement of the Environment scale (at 6, 18 & 36 months); Activities with child (at 6, 18, 36 months); Home Learning Environment (at 48 months); Material Deprivation scale (at 18 months); Framingham Safety Survey (at 6, 18, & 48 months); Family Environment Scale (at 12 &36 months); Difficult Life Circumstances (18 & 36 months); Neighbourhood Quality Evaluation Scale (at 36 months); Family Routine Inventory (at 36 months).  5. Parent health: physical health outcomes (self-reported) (at baseline, 6, 12, 18, 24, 36, 48 months); personality (Tem-Item Personality Inventory (baseline), Rosenberg self-esteem (at baseline12, 18, 48 months) Pearlin Self-Efficacy Scale (at baseline,12 & 48 months) ; Vulnerable Attachment Style Questionnaire (baseline); Considerations of Future Consequences (at baseline & 24 months); Future Outlook Inventory (at 12, 36 months); Self-control (at 18 months);  mental health outcomes (Edinburgh Postnatal Depression Scale at 6, 18, 24, 36, & 48); WHO-5 Index (at baseline, 6, 12, 36, 48) 6. Social support and service use: Level from support from various people (at 6, 18, 24, 36, & 48 months); community integration (at 6, 12, 18, 24, 36, 48 months); use of services (at 6, 18, 36 months); partner satisfaction (at 6, 12, 24, 48 months); Maternal Social Support Index ( at 18 & 36 month); Family Quality of Life (at 36 months); Relationship Quality Index (at 36 months). |

# 2. INTRODUCTION AND RATIONALE

Deprivation early in life has multiple long term consequences for both the individual and society in general. The consequences of being raised in disadvantaged circumstances are significant, as socioeconomic inequalities in children’s health and development emerge early and increase over time (Najman *et al*., 2004; Shonoff and Philipps, 2000). Growing up in poverty can affect a child’s early skill development leading to greater vulnerability at school entry (Duncan and Brooks-Gunn, 1997), poorer cognitive skills (Stipek and Ryan, 1997), less developed social skills (Janus and Duku, 2007), as well as more emotional and behavioural problems (McLoyd, 1998). In addition, such early developmental difficulties can also affect major long term public and social policy issues such as academic achievement (Raver, 2003), employment (Rouse, Brooks-Gunn and McLanahan, 2005), teenage pregnancy, and psychological well-being (Brooks-Gunn, 2003).

Such deprivation is intergenerational in nature and is difficult to eradicate. Remediation policies are the most common method for addressing social inequalities, yet evidence suggests that they are both costly and less effective than preventative policies (Carneiro and Heckman, 2003). An increasing body of evidence finds that targeted, early interventions aimed at at-risk children and their families can reduce socioeconomic disparities in children’s capabilities (see Sweet and Appelbaum, 2004 for a review). While a number of individual studies have found home visiting programmes to generate significant and positive short and long term outcomes (e.g. Olds et al., 1999;), a meta-analysis of home visiting programmes evaluated by experimental design found significant variation in programme effectiveness across studies (Sweet and Appelbaum, 2004). Much of this evidence is US based and there is a clear lack of research on the effects of early intervention in countries with different social and cultural contexts such as Ireland.

Investment in early intervention programmes is efficient from both biological and economic perspectives. Intervening early in life, when children are at their most receptive stage of development, has the potential to permanently alter their brain development and subsequent developmental trajectories (Halfon, Shulman, and Hochstein, 2001). Early intervention is also economically efficient. Research on US intervention programmes has demonstrated high rates of return such that the individual and societal benefits accrued from intervening early typically outweigh the costs (Karoly, Kilburn, and Cannon, 2005).

This study describes a randomised control trial (RCT) evaluation of a preventative programme which aims to improve the life outcomes of socioeconomically disadvantaged children. The programme will operate in several disadvantaged communities of Dublin with above national average rates of unemployment, early school leavers, lone parent households and social housing (Census, 2006). The *Preparing for Life* (PFL) programme works with families from pregnancy until school entry in order to promote positive child development through improved parental behaviour and social support.

# 3. AIMS, HYPOTHESIS and OBJECTIVES

**3.1 Aim**

To test the effectiveness of the Preparing for Life (PFL) programme at improving the school readiness skills and life outcomes of socioeconomically disadvantaged children.

**3.2 Primary hypothesis**

It is hypothesised that the children participating in Preparing for Life programme will be significantly better prepared for school than those in the low treatment group. The null hypothesis is that there will be no difference in school readiness skills between the high treatment and low treatment groups; this may arise if the intervention is not effective or if there is substantial contamination between groups.

**3.3 Secondary hypotheses**

To test the effectiveness of the Preparing for Life (PFL) programme at improving parental skills and the quality of the home environment.

# 4. OBJECTIVES

- To undertake a randomised controlled trial to evaluate whether, compared to the low treatment group, the PFL home visiting programme and group Triple P leads to improved school readiness skills among 4/5 year old children who are socioeconomically vulnerable.

- To use a quasi-experimental design to evaluate whether, compared to the external comparison group, the low treatment supports leads to improved school readiness skills among 4/5 year old children who are socioeconomically vulnerable.

# 5. OUTCOME MEASURES

**5.1 Primary endpoint**

*School Readiness Skills*

1. **Cognitive development**: measured using Ages and Stages Questionnaire (at 6, 12, 18, 24, 36, 48 months); Developmental Profile 3 (at 12, 18, 24, 36, 48); British Ability Scales (at 48 months); Executive functioning & delay of gratification (48 months) ); Early Development Instrument- Short Form (at school entry).
2. **Physical health and motor skills**: measured using Ages and Stages Questionnaire (at 6, 12, 18, 24, 36, 48 months); hospital records (at 48 months); parent-reported child health (at 6, 12, 18, 24, 36, 48); Early Development Instrument- Short Form (at school entry).
3. **Socio-emotional development**: measured using Temperament and Atypical Behaviour Scale (at 12 months); Difficult temperament (at 6 & 12 months); ASQ-Socio-Emotional scale (at 6, 12, 18, 24, 36, 48 months); Brief Infant Toddler Social and Emotional Assessment (at 12, 18 24, 36 months); Early Development Instrument- Short Form (at school entry).
4. **Behavioural skills**: measured using Temperament and Atypical Behaviour Scale (at 12 months); Child Behaviour Checklist (at 18, 36, 48 months); Peer Problems and Prosocial Behaviour (at 48 months); Early Development Instrument- Short Form (at school entry).
5. **Language development and emergent literacy**: measured using Mac-Arthur Bates Communicative Development Inventories (at 12, 18, 24 months); Ages and Stages Questionnaire (at 6, 12, 18, 24, 36, 48 months); British Ability Scales (at 48 months); Early Development Instrument- Short Form (at school entry).

**5.2 Secondary endpoints**

1. **Birth outcomes:** maternity hospital records at birth (birth weight, gestational age, prematurity, Apgar score).
2. **Labour outcomes:** maternity hospital records at birth (instrumental delivery, caesarean section- elective, emergency).
3. **Parenting skills:** Adult Adolescence Parenting Inventory (at baseline &12 months); Knowledge of Infant Development (at baseline & 12 months); Parental locus of control (at 6 months); Condon Maternal Attachment Scale (at 6 & 24 months); Parenting Stress Index (at 6, 24 & 48 months); Parenting Daily Hassles Scale (at 18, 36 & 48 months).; Maternal Separation Anxiety (at 18 months); Parental Cognitions and Conduct Towards Infant Scale (at 6 & 24 months); Parenting Styles and Dimensions Questionnaire (at 36 & 48 months); Parental Acceptance -Rejection Questionnaire (at 36 months).
4. **Quality of the home environment:** Home Observation for Measurement of the Environment scale (at 6, 18 & 36 months); Activities with child (at 6, 18, 36 months); Home Learning Environment (at 48 months); Material Deprivation scale (at 18 months); Framingham Safety Survey (at 6, 18, & 48 months); Family Environment Scale (at 12 &36 months); Difficult Life Circumstances (18 & 36 months); Neighbourhood Quality Evaluation Scale (at 36 months); Family Routine Inventory (at 36 months).
5. **Parent health:** physical health outcomes (self-reported) (at baseline, 6, 12, 18, 24, 36, 48 months); personality (Tem-Item Personality Inventory (baseline), Rosenberg self-esteem (at baseline12, 18, 48 months) Pearlin Self-Efficacy Scale (at baseline,12 & 48 months) ; Vulnerable Attachment Style Questionnaire (baseline); Considerations of Future Consequences (at baseline & 24 months); Future Outlook Inventory (at 12, 36 months); Self-control (at 18 months); mental health outcomes (Edinburgh Postnatal Depression Scale at 6, 18, 24, 36, & 48); WHO-5 Index (at baseline, 6, 12, 36, 48).
6. **Social support and service use:** Level from support from various people (at 6, 18, 24, 36, & 48 months); community integration (at 6, 12, 18, 24, 36, 48 months); use of services (at 6, 18, 36 months); partner satisfaction (at 6, 12, 24, 48 months); Maternal Social Support Index ( at 18 & 36 month); Family Quality of Life (at 36 months); Relationship Quality Index (at 36 months).

**6. STUDY Design**

The study is a two-arm researcher-blind, randomised controlled trial (RCT), to test the effectiveness of the PFL home visiting programme and group Triple P for pregnant women from socioeconomically disadvantaged communities. The trial will involve 300 families in total. 200 will be randomly allocated into one of two groups:

(1) **High treatment group**, who will receive the PFL intervention (described below) (n=100)

(2) **Low treatment group** (n=100)

A third external comparison group will also be recruited from another similar community

(3) **External comparison group** (n=100).

**7. Trial Intervention**

**7.1 High treatment intervention**

*PFL* is a community-based home visiting programme (HVP) which aims to improve children’s school readiness skills by intervening during pregnancy and working with families until the children start school at age 4/5 years. The high treatment group will receive twice monthly home visits, lasting approximately one hour, delivered by mentors from a cross-section of professional backgrounds such as psychologists and family support specialists. Mentors will receive extensive training prior to programme implementation and monthly supervision thereafter to ensure fidelity to the programme model. Each family will be assigned the same mentor over the course of the intervention where possible. The mentors will use role modelling, demonstration, coaching, discussion, encouragement, and feedback to deliver the intervention, as well as direct interactions with the child in the presence of the parent. The aim of the visits are to support and educate the parents on key child rearing issues including the identification of developmental milestones and appropriate parenting practices that promote the children’s health, and cognitive and non-cognitive development. Each visit will be guided by a set of *PFL* ‘Tip Sheets’ which are based on pre-existing governmental and local non-governmental organisations’ recommendations, and present best-practice information on pregnancy, parenting, and child health and development. There are three sets of age-specific Tip Sheets - pre-birth-12 months, 1-2 years, and 2-4 years. There are approximately 150 *PFL* Tip Sheets as part of the programme’s curriculum. The mentors will be able to choose when to deliver the Tip Sheets within these specific time periods based on the age of the child and the needs of the family. The Tip Sheets will be given to the participants at the end of each visit to keep as an on-going resource.

The high treatment group will also be invited to participate in an additional parenting course (Triple P Positive Parenting Program; Sanders et al., 2003) when their children are between 2 and 3 years old i.e., after they have completed the 24-month assessment. Triple P promotes healthy parenting practices and positive parent-child attachment. The intervention will involve the delivery of Group Triple P which consists of 5 two-hour group discussion sessions and 3 individual phone calls facilitated by the mentors.

**7.2 Low treatment intervention**

Both the high and low treatment groups will receive some common supports including developmental materials and book packs. The developmental packs will consist of materials such as a baby gym, food utensils, safety items and an assortment of developmental toys. Both groups will also be encouraged by letter and text message to attend public health workshops on stress management and healthy eating which are already taking place in the community. The low treatment group will also have access to a support worker who can help them avail of community services if needed, while this function will be provided by the mentors for the high treatment group.

**7.3 External comparison group intervention**

The external comparison group, which will be selected from a socio-demographically similar community, will receive care-as-usual only. Care as usual, which is available to all pregnant women and infants in Ireland, is as follows: Expectant mothers are provided with an initial family doctor (G.P.)/obstetrician appointment at 12 weeks and a further 5 examinations for first time mothers and 6 for subsequent pregnancies. Antenatal classes are provided by local public maternity hospitals free of charge. Following birth, a G.P. examination is carried out for the baby at 2 weeks and mother and baby at 6 weeks. The mother is entitled to free in-patient, out-patient and accident and emergency/casualty services in public hospitals in respect of the pregnancy and the birth and is not liable for any hospital charges. In addition, checks by a public health nurse are generally carried out in the home in the weeks after birth and when the infant is 9, 18, and 24 months, but they are not mandatory. A schedule of immunizations in provided free of charge at birth, 2, 4, 6, 12, and 13 months

**8. Flow Chart**

**9. STUDY POPULATION**

All pregnant women from the PFL catchment communities (defined by the Northside Partnership who will manage the intervention) are eligible to participate.

**9.1 Eligibility criteria**

**Inclusion criteria**

1. Women (age 16+) pregnant between 2008 and 2010
2. Residing in the PFL catchment area
3. Willing to be assigned to either of the study intervention groups
4. Written informed parental/carer consent

**Exclusion criteria**

1. Under the age of 16
2. Not pregnant
3. Not residing in the PFL catchment area
4. No written consent provided.

**10. study Procedures**

**10.1 Recruitment**

Recruitment into the study will occur through one of two sources: 1) in the maternity hospital at the first booking visit or 2) self-referrals in the community. The recruitment process will involve substantial interactions and collaboration with the maternity hospitals in order to identify eligible women in a confidential manner. The hospital administrative staff will send the UCD evaluation team a weekly list stating the number of women from the *PFL* catchment area who are scheduled to have their first booking visit at the hospital the following week. The list will include the time and date of the visit, but for confidentiality reasons, no names or contact information will be included. The hospital staff will also flag the files of any eligible women on their computer system. By doing this, an alert will appear on the computer screen when the eligible women are booked into the clinic in the Outpatient Department (OPD). When this alert appears, the OPD staff will give the eligible women a *PFL* flier describing the programme and introduce them to the *PFL* recruiter who will be present in the waiting room of the clinic. The recruiter will then briefly explain the programme to the eligible participants and ask for their contact details. If the eligible participant is interested in learning more about the programme, the recruiter will take their contact details. Next, the recruiter will ring the potential participant later that day to set up a recruitment appointment to take place in the village centre or the family home. During the recruitment appointment, the recruiter will describe the study in detail and bring the eligible participant through the information and consent form. If the eligible participant agrees to join the study she will be asked to sign the consent form. The same process will be used for all 3 groups (high treatment, low treatment, and comparison).

**10.2 Blinding**

Researchers assessing the study outcomes will be blinded to the randomised allocation. Participants (i.e. parents) will be informed of their randomised allocation.

**10.3 Randomisation**

Participants will be randomised after informed consent is obtained using an unconditional probability randomisation procedure. No stratification of block techniques will be used. A computerised programme will be used to create an array of numbers populated with a one or zero equal to the required sample size. This array will be shuffled using a random number generator to randomly assign the numbers a location in the array. To ensure randomisation is not compromised the participant will press a key on a computer which randomly allocated her treatment group assignment. Once assignment is completed, an automatic email will be generated which includes the participant’s unique ID number and assignment condition. This email will be automatically sent to the *PFL* programme manager and the evaluation manager. This method will be used to ensure that the recruiter has no influence on the treatment assignment. Thus, if any attempts to reassign participants from one group to another group, by either directly changing the database or repeating the randomisation procedure, a second email will be generated to automatically highlight this intentional subversion.

# 10.4 Assessments

**Timing:** Data will be collected from both the programme participants (both high and low treatment groups) and the external comparison group within the same time period. Baseline data will be collected after confirmation of informed consent/randomisation assignment and before the programme begins. Subsequently, data will be collected when the infants are 6, 12, 18, 24 months of age and then each subsequent year (36 & 48 months) until the study child starts school (at age 4/5). Records from the maternity hospitals will be collected after the birth of the child and records from the children’s hospital will be collected when the child is at least 4 years old. It is important to collect data more regularly during the early stages of the child’s development as the evidence based on US interventions e.g. the Early Head Start programme, found that interventions have the greatest impact between 12 and 18 months. In addition, more data points will help track developmental trajectories. In total, eight waves of data will be collected. As the data collection will be sequential, each family will be interviewed when the child is the same age.

**Informant*:*** A combination of data capture methods will be used to produce optimum quality data. The primary informant at household level will be the mother through CAPI interview. The father, if available and living in the household, will also be required to participate in a shorter self-administered interview. Based on a scoping survey we estimate that about 50% of children will have biological parents living apart. If contact details are available from the mother, the absent biological father will be contacted and asked to complete the self-administered questionnaire. Data will also be collected on any siblings in the household. These data are crucial for evaluation of the RCT component, as given the design of the programme a pre-test (i.e. a pre-test measures outcomes prior to service delivery to facilitate comparison with post programme outcomes), is not possible. Therefore examining the key outcomes of the siblings of the PFL children before the programme begins will allow us to determine whether the parents’ behaviour, and its subsequent effect on the PFL children, differs before and after PFL. Additionally, data will be collected from junior infant school teachers (using the early development Instrument).

**Interviewers*:*** All interviews will be conducted by trained research assistants. The researchers will receive specialised training from our training partners, the National Centre for Social Research (NatCEN), to minimise interview bias. Specialised training will be required for this study as it involves working with children and parents on sensitive issues and standardising measurements of height and weight to minimise inter and intra-observer variation.

**Assessments & Contact Time*:*** Appointments to schedule the data collection and assessment interviews will be made in advance by telephone and will be considerate of the respondents’ needs and availability. Family contact time for data collection will be confined to about 2 hours per case, with a natural break in between. The 2 hours duration for data collection has been widely established in other studies as the optimal duration for maximizing the amount of data collected and minimizing participant fatigue and attrition from the study. Allowing sufficient time is necessary as it is important for families to feel that they are being carefully listened to by the interviewers if the goal is to assess their current life situation, their needs, and their perceptions about PFL services they are receiving.

**Computer-Aided Personal Interviews *(CAPI):*** Data collection will be achieved using Computer-Aided Personal Interviews (CAPI) in the home setting. Using this method, data entry, editing, and verification procedures are programmed into a laptop computer. The interviewer – or the respondent – records answers to each question using the keyboard or screen. Automatic routing ensures that there are no interviewer-driven errors in asking the correct question. One advantage of the home setting is that it provides access to fathers who appear to be more willing to participate if data collection occurs conveniently in the home. Another advantage of the home setting is that measures of parenting skills and the home environment can be obtained. This is particularly useful since the intervention focuses on parenting practices.

**Self-completed Interviews*:*** At the time of the interview with the principal informant in the household (usually the mother), it is planned to give all fathers present a self-completion questionnaire. The data will be entered onto a computer as rapidly as possible after completion. In addition, as the children advance in age, interviews will be conducted with them, so as to ensure that the voice and mind of the child is captured in as an informative but unobtrusive a way as possible.

Justification of Assessments*:* In order to be ready for school, children need to have attained several physical, cognitive and social developmental milestones. There are numerous factors determining a child’s level of school readiness, but the risks are correlated and tend to be concentrated in low socioeconomic status families. Maternal education, educational quality of the home environment, and socioeconomic status (SES) appear to be the best predictors of children’s early grade school success (Horvat & Svetina, 1993; Ricciuti, 1999; Ketterlinus, Henderson, Lamb, 1991; Kinard & Reinherz, 1987). Children from very low SES backgrounds are more likely than other children to exhibit deficits in their verbal abilities, visual-motor abilities, and motor abilities (Costeff & Kulikowski, 1996). Such risks, which many of the PFL children may face, begin to act as early as the prenatal life period, with maternal health behaviors during pregnancy. Mothers who live in economically deprived conditions are more likely to experience stress and to consume alcohol and nicotine during pregnancy. In the post-natal period, parental behaviours and attitudes, such as expectations and parenting practices in early childhood, as well as the effective bond between parents and their children, have been found to predict early grade school success. In addition, disadvantaged children tend to experience lower quality of education in the home (e.g. lower number of books, less frequent reading by parents) which is predictive of school readiness (Clarke and Kurtz-Costes (1997). Importantly, crèche and preschool experiences can enhance the cognitive development of low-SES children (Geoffroy et al., 2007). Thus, school readiness and early academic achievement are determined by a number of children’s characteristics and environmental influences that come together during first few years of life. Consequently, assessment of the impact of the PFL intervention and of the mechanism of action will carefully examine the social, physical and cognitive development of the child, his/her family background, home environment, and preschool environment.

Survey Instruments*:* Table 1 below lists the complete set of instruments which will be collected over the 8 data assessment point. The assessments will be divided into two part – the primary child outcome measures and the secondary parent outcome measures. The parent information will include 1) Demographic/Household, 2) Employment/Income, 3) Pregnancy, 4) Parenting/Neglect, 5) Maternal Social Support, 6) Maternal Socio-emotional Functioning, 7) Maternal Health, 8) Maternal Drug/Alcohol Use, 9) the Home Environment, 10) Breastfeeding, 11) Childcare Decisions, 12) Maternal Cognitive Assessment, 13) General Questions, 14) Programme Satisfaction, 15) Open-ended Questions, 16) Sibling Behaviour. The child information will include 1) Child Development and 2) Child Health. The specific instruments for each domain are listed in Table 1.

Providing Feedback to Families after Assessment*:* The interviewer will end each interview sessions with a debriefing in which the interviewer will thank the respondents for their time and ask them if they have any questions and how they felt the interview went. If an assessment reveals that a child is above a certain level of clinical risk, in terms of developmental delay for example, we will inform the parents of this and write a letter referring them to the appropriate agency. It should be noted that this may bias the results of the impact evaluation if a significant number of children are at this level, however it is essential to maintain a balance between scientific rigor and ethical considerations.

**Maintaining Contact with Families Between Assessments*:*** The research team will maintain contact with the PFL families and the external comparison group between assessments using the Database Management System (DBMS) to enable contact with, and tracking of, participants. This system will be used to track the consent status and any contacts between the researchers and the PFL participants, as well as change of address or any other factors that may affect the researcher’s ability to carry out the evaluation. A lo-call number will also be maintained in order for families to contact the research team. In addition, a programme newsletter which talks about the importance of evaluating the programme and highlights some basic data findings may help the participant families to better connect with the research side of the programme.


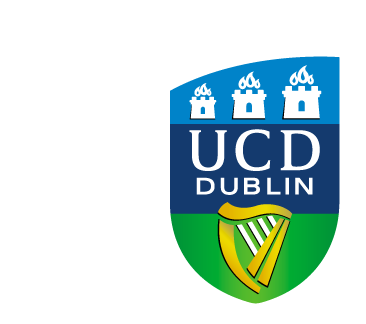

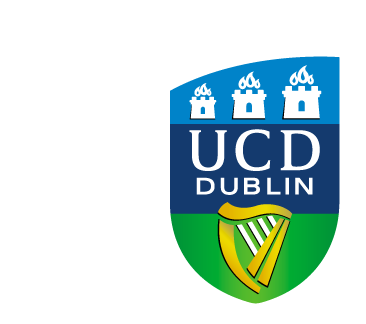
***Table 1***

***Preparing for Life* (*PFL*) Survey Content for All Waves of Data Collection**

**Parent/Family Survey Content**

| **Category** | **Measure/Information Obtained** | **BL** | **3** | **6** | **12** | **18** | **24** | **36** | **48** |
| --- | --- | --- | --- | --- | --- | --- | --- | --- | --- |
|  | Father Questionnaire Distributed | X |  | X |  |  |  |  |  |
| **Interview Details** | RA Initials |  |  |  | X | X | X | X | X |
|  | ID number | X | X | X | X | X | X | X | X |
|  | Date of Interview | X | X | X | X | X | X | X | X |
|  | Child’s Name (note that this is not permanently recorded in datafile) |  |  | X | X | X | X | X | X |
|  | Multiple Birth |  |  | X | X | X | X | X | X |
|  | Location of Interview |  |  | X | X | X | X | X | X |
|  | Survey Method |  |  |  | X | X | X | X | X |
|  | Child Present |  |  |  |  |  |  | X | X |
| **Demographic/**  **Household Information** | Maternal Age | X |  |  |  |  |  |  |  |
|  | Maternal Ethnic Group | X |  |  |  |  |  |  |  |
|  | Maternal Age at Birth of her First Child | X |  |  |  |  |  |  |  |
|  | Indicator for whether Mother was in Special/Remedial Education | X |  |  |  |  |  |  |  |
|  | Maternal Grandmother Age & Education | X |  |  |  |  |  |  |  |
|  | Maternal Grandfather Age & Education | X |  |  |  |  |  |  |  |
|  | Father’s Age | X |  |  |  |  |  |  |  |
|  | Indicator If Father was in Special/Remedial Education | X |  |  |  |  |  |  |  |
|  | Paternal Grandmother Age & Education | X |  |  |  |  |  |  |  |
|  | Paternal Grandfather Age & Education | X |  |  |  |  |  |  |  |
|  | Household Composition | X |  | X | X | X | X | X | X |
|  | Number of Mother’s Biological Children | X |  | X |  |  |  |  |  |
|  | Mother’s Relationship/Marital Status | X |  | X | X | X | X | X | X |
|  | Indicator if Partner is Biological Father |  |  | X | X | X | X | X | X |
|  | Maternal Highest Education Level | X |  | X |  |  |  |  |  |
|  | Age Mother Left Full-time Education | X |  | X |  |  |  |  |  |
|  | Mother’s Intention to Remain in School (if still in full-time education) | X |  | X |  |  |  |  |  |
|  | Maternal Education: Continuation of Education/Additional Courses Taken |  |  |  |  |  | X | X | X |
|  | Maternal Literacy Problems (in daily life) | X |  |  |  |  |  |  |  |
|  | Maternal Maths Problems (in daily life) | X |  |  |  |  |  |  |  |
| **Category** | **Measure/Information Obtained** | **BL** | **3** | **6** | **12** | **18** | **24** | **36** | **48** |
|  | Maternal Home Ownership Status | X |  | X |  |  |  | X |  |
|  | Mother’s Relationship with and the Involvement of the Biological Father | X |  | X | X | X |  |  |  |
|  | Father’s Highest Education Level | X |  | X |  |  |  |  |  |
|  | Age Mother’s Partner Left Full-time Education | X |  | X |  |  |  |  |  |
| **Employment/ Income Information** | Maternal Grandfather’s Main Occupation | X |  |  |  |  |  |  |  |
|  | Maternal Grandmother’s Main Occupation | X |  |  |  |  |  |  |  |
|  | Mother’s Maternity Leave |  |  | X | X |  |  |  |  |
|  | Mother’s Work Status | X |  | X | X | X | X | X | X |
|  | Mother’s Occupation | X |  | X | X | X | X | X | X |
|  | Mother’s Work Hours | X |  | X | X | X | X | X | X |
|  | Mother’s Wage | X |  | X | X | X | X | X | X |
|  | Maternal Job Stability |  |  |  | X | X | X | X | X |
|  | Mother’s Unemployment Info (if applicable) | X |  | X | X | X | X | X | X |
|  | Indicator for Social Welfare Payments to Household | X |  | X | X |  |  |  |  |
|  | Indicator for Social Welfare Payments to Household – Detailed |  |  |  |  | X | X | X | X |
|  | Household Weekly Income | X |  | X | X | X | X | X | X |
|  | Family Finances |  |  |  |  |  |  | X | X |
|  | Perception oF Financial Difficulty/Economic Perceptions | X |  | X | X | X | X | X | X |
|  | Mother’s Saving Habits | X |  | X | X | X | X | X | X |
|  | Partner’s Work Status | X |  | X | X | X | X | X | X |
|  | Partner’s Occupation | X |  | X | X | X | X | X | X |
|  | Partner’s Work Hours | X |  | X | X | X | X | X | X |
|  | Partner’s Wage | X |  | X | X | X | X | X | X |
|  | Partner’s Job Stability |  |  |  | X | X | X | X | X |
|  | Father’s Unemployment Info (if applicable) | X |  | X | X | X | X | X | X |
| **Pregnancy** | Week of Pregnancy | X |  |  |  |  |  |  |  |
|  | Child Due Date | X |  |  |  |  |  |  |  |
|  | Maternity Hospital Mother Attending | X |  |  |  |  |  |  |  |
|  | Week Pregnancy Confirmed | X |  |  |  |  |  |  |  |
|  | Week of First Antenatal Visit | X |  |  |  |  |  |  |  |
|  | Indicator of Mother’s Intention to take Antenatal Classes | X |  |  |  |  |  |  |  |
|  | Indicator of Whether Pregnancy was Planned | X |  |  |  |  |  |  |  |
|  | Mother’s Reaction to Pregnancy | X |  |  |  |  |  |  |  |
|  | Mother’s Family’s Reaction to Pregnancy | X |  |  |  |  |  |  |  |
|  | Mother’s Health Supplement Use (separate questions for multi-vitamins, folic acid, iron, calcium) Before and During Pregnancy | X |  |  |  |  |  |  |  |
| **Category** | **Measure/Information Obtained** | **BL** | **3** | **6** | **12** | **18** | **24** | **36** | **48** |
|  | Number of Health Visits and Checkups During Pregnancy | X |  |  |  |  |  |  |  |
|  | Number of Previous Pregnancies (incl. those not to full term) | X |  |  |  |  |  |  |  |
|  | Maternal Age at Previous Pregnancies (incl. those not to full term) | X |  |  |  |  |  |  |  |
|  | Mother’s Birth Control Practices | X |  |  | X | X |  | X | X |
| **Parenting/**  **Neglect** | Assessment of Parenting Risks: *Adult-Adolescent Parenting Inventory II (AAPI-II)* | X |  |  | X |  |  |  |  |
|  | Mother’s Knowledge on Parenting Practices and Child Development Norms: *Knowledge of Infant Development Inventory (KIDI-Short Form)* | X |  |  | X |  |  |  |  |
|  | Children and Their Parents: *Parental Locus of Control* |  |  | X |  |  |  |  |  |
|  | Maternal Attachment: *Condon Maternal Attachment Scale* |  |  | X |  |  | X |  |  |
|  | Feelings about Parenting: *Parenting Stress Index* |  |  | X |  |  | X |  | X |
|  | Parenting Stress: *Parenting Daily Hassles Scale* |  |  |  |  | X |  | X | X |
|  | Separation Anxiety: *Maternal Separation Anxiety Scale (General Separation Anxiety Scale only)* |  |  |  |  | X |  |  |  |
|  | Maternal Perceptions and Behaviours: *Parental Cognitions and Conduct Towards Infant Scale (PACOTIS)* |  |  | X |  |  | X |  |  |
|  | Parenting Behaviour: *Parenting Styles and Dimensions Questionnaire* |  |  |  |  |  |  | X | X |
|  | Parental Acceptance: *Parental Acceptance-Rejection Questionnaire* |  |  |  |  |  |  | X |  |
|  | Child Protective Services Involvement |  |  |  | X |  | X | X | X |
|  | Parenting Resources |  |  |  | X |  |  | X |  |
|  | Activities with Baby/Child |  |  | X |  | X |  | X |  |
|  | Activities with Baby/Child: *Home Learning Environment (HLE)* |  |  |  |  |  |  |  | X |
|  | Parental Attitudes Toward Education |  |  |  |  |  |  | X |  |
|  | Parental Monitoring of TV |  |  |  |  |  |  | X | X |
| **Maternal Social Support** | Level of Support Mother receives from Family, Partner, Friends, Neighbours, Work Colleagues, and *PFL* Programme | X |  | X |  | X | X | X | X |
|  | Mother’s Comfort Level Asking for Help | X |  |  |  |  |  |  |  |
|  | Mother’s Frequency of Meeting with Friends/Relatives | X |  | X | X |  | X | X | X |
|  | Mother: Number of Neighbours Know Personally | X |  |  |  |  |  | X |  |
|  | Mother’s Participation in Organizations | X |  |  |  |  | X |  |  |
|  | Frequency of Community Services Use (63 services) | X |  | X |  | X |  | X |  |
|  | Child Contact with/help from Grandparents |  |  | X | X |  |  |  |  |
|  | Mother Number of Neighbours with Child |  |  | X | X |  | X | X | X |
|  | Mother Contact with Other People in Programme |  |  | X | X | X |  | X | X |
|  | Mother Share Programme Information |  |  | X | X | X | X | X | X |
|  | Influences Parenting Decisions |  |  | X | X | X | X | X | X |
| **Category** | **Measure/Information Obtained** | **BL** | **3** | **6** | **12** | **18** | **24** | **36** | **48** |
| **Maternal Social Support**  ***(continued)*** | Father’s Involvement with Baby/Child |  |  | X | X | X | X | X | X |
|  | Satisfaction with Father Involvement |  |  | X | X |  | X |  | X |
|  | Partner’s Involvement with Baby/Child (if different than father) |  |  | X | X | X | X | X | X |
|  | Satisfaction with Partner’s Involvement (if different than father) |  |  | X | X |  | X |  | X |
|  | Father Maintenance |  |  |  | X | X | X | X | X |
|  | Maternal Social Support: *Maternal Social Support Index (adapted)* |  |  |  |  | X |  | X |  |
| **Maternal Socio-Emotional Functioning** | Personality: *Ten-Item Personality Inventory (TIPI)* | X |  |  |  |  |  |  |  |
|  | Indicator for Postnatal Depression in Previous Pregnancies | X |  |  |  |  |  |  |  |
|  | Mother’s self-reported DX of Postnatal Depression in past 6 months |  |  | X |  |  |  |  |  |
|  | Mother’s self-reported Prescription for Postnatal Depression |  |  | X |  |  |  |  |  |
|  | Maternal Postnatal Depression (6 months): *Edinburgh Postnatal Depression Scale* |  |  | X |  |  |  |  |  |
|  | Maternal Postnatal Depression (past 7 days): *Edinburgh Postnatal Depression Scale* |  |  | X |  | X | X | X | X |
|  | Maternal Self-Esteem: *Rosenberg Self-Esteem scale* | X |  |  |  | X | X |  | X |
|  | Maternal Self-Efficacy: *Pearlin Self-Efficacy scale* | X |  |  | X |  |  |  | X |
|  | Maternal Attachment Style : *Vulnerable Attachment Style Questionnaire* | X |  |  |  |  |  |  |  |
|  | Consideration of Future: *Consideration of Future Consequences Scale* | X |  |  |  |  | X |  |  |
|  | Maternal Psychological Well-Being: *WHO-5 Index* | X |  | X | X |  |  | X | X |
|  | Time Perspective Taking: *Future Outlook Inventory (FOI)* |  |  |  | X |  |  | X |  |
|  | Self-Control: *Baumeister Self-Control Measure* |  |  |  |  | X |  |  |  |
|  | Social Desirability: *Social Desirability Scale-17* |  |  |  |  |  | X |  |  |
|  | Maternal Antisocial Behaviour |  |  |  |  |  | X |  |  |
|  | Family Quality of Life: *Beech Center Family Quality of Life Scale* (exclude physical disability subscale) |  |  |  |  |  |  | X |  |
|  | Romantic Relationship Quality: *Relationship Quality Index* |  |  |  |  |  |  | X |  |
| **Maternal Health** | Mother’s Health as a Child | X |  |  |  |  |  |  |  |
|  | Mother’s Height & Pre-pregnancy Weight | X |  |  |  |  |  |  |  |
|  | Mother’s Current Weight |  |  | X | X |  |  |  |  |
|  | Mother’s General Health Status | X |  |  | X | X | X | X | X |
|  | Indicator for Mother has Long Term Illness | X |  |  |  |  |  |  |  |
|  | Existence and Type of Mother’s Physical Medical Conditions | X |  |  |  |  |  |  |  |
|  | Existence and Type of Mother’s Psychological Health Conditions | X |  |  |  |  |  |  |  |
|  | Indicator of whether Mother taking Prescribed Medication | X |  |  |  |  |  |  |  |
|  | Existence and Type of Family Psychological Conditions | X |  |  |  |  |  |  |  |
|  | Indicator for whether Mother has/ever had Medical Card | X |  |  |  | X |  |  |  |
|  | Indicator for whether Mother has/ever had GP Visit Card | X |  |  |  | X |  |  |  |
|  | Indicator for whether Mother has Private Health Insurance | X |  |  |  |  |  |  |  |
| **Category** | **Measure/Information Obtained** | **BL** | **3** | **6** | **12** | **18** | **24** | **36** | **48** |
| **Maternal Health**  ***(continued)*** | Mother’s Diet/Eating Habits | X |  |  | X |  |  |  |  |
|  | Mother’s Exercise Habits | X |  |  | X |  |  |  |  |
|  | Mother’s Health Services Use in last 12 months | X |  |  |  | X |  | X |  |
|  | Maternal Health since Birth of Baby |  |  | X |  |  |  |  |  |
|  | Maternal Health During Pregnancy |  |  | X |  |  |  |  |  |
|  | Mother’s Breathing |  |  | X |  |  |  |  |  |
|  | Mother’s GP Visits |  |  | X | X | X | X | X | X |
|  | Family Planning |  |  |  | X | X | X | X | X |
| **Maternal Drug/ Alcohol Use** | Mother’s Cigarette Use Before Pregnancy | X |  |  |  |  |  |  |  |
|  | Mother’s Reasons for Change in Cigarette Use During Pregnancy | X |  |  |  |  |  |  |  |
|  | Mother’s Alcohol Use Before Pregnancy (quantity & type) | X |  |  |  |  |  |  |  |
|  | Mother’s Reasons for Change in Alcohol Use During Pregnancy | X |  |  |  |  |  |  |  |
|  | Mother’s Drug Use Before Pregnancy (quantity & type) | X |  |  |  |  |  |  |  |
|  | Mother’s Reasons for Change in Drug Use During Pregnancy | X |  |  |  |  |  |  |  |
|  | Mother’s Drug Use During Pregnancy | X |  | X |  |  |  |  |  |
|  | Mother’s Change in Cigarette Use During Pregnancy | X |  | X |  |  |  |  |  |
|  | Mother’s Alcohol Use During Pregnancy | X |  | X |  |  |  |  |  |
|  | Mother’s Change in Alcohol Use During Pregnancy | X |  | X |  |  |  |  |  |
|  | Mother’s Drug Use During Pregnancy | X |  | X |  |  |  |  |  |
|  | Mother’s Change in Drug Use During Pregnancy | X |  | X |  |  |  |  |  |
|  | Current Cigarette Use |  |  | X | X | X | X | X | X |
|  | Indicator if Household Members Smoke in same room as Mother/Baby/Child | X |  | X | X |  |  |  |  |
|  | Mother’s Current Alcohol Use |  |  | X | X | X | X | X | X |
|  | Mother’s Current Drug Use |  |  | X | X | X | X | X | X |
| **Breastfeeding** | Indicator for Mother Breastfed in Previous Pregnancies | X |  |  |  |  |  |  |  |
|  | Mother’s Intention to Breastfeed New Baby | X |  |  |  |  |  |  |  |
|  | Intergenerational Breastfeeding |  |  | X |  |  |  |  |  |
|  | Current Breastfeeding Practices |  |  | X | X | X | X |  |  |
| **Environment** | Household Material Deprivation Assessment: *EU-SILC Survey* | X |  |  |  | X |  |  |  |
|  | Indicators of Household Domestic Social/Emotional Risk | X |  |  | X |  | X |  | X |
|  | Safety: *Injury Prevention Program Framingham Safety Survey* |  |  | X |  | X |  |  | X |
|  | Home Environment: *Home Observation for Measurement of the Environment (HOME)* + *Supplement to the HOME for Impoverished Families (SHIF)* |  |  | X |  | X |  | X |  |
|  | Family Environment: *Family Environment Scale (FES)* |  |  |  | X |  |  | X |  |
|  | Life Circumstances: *Difficult Life Circumstances* |  |  |  |  | X |  | X |  |
|  | Mother’s Satisfaction with Neighbourhood | X |  |  |  |  |  | X |  |
| **Category** | **Measure/Information Obtained** | **BL** | **3** | **6** | **12** | **18** | **24** | **36** | **48** |
| **Environment**  ***(continued)*** | Neighbourhood Quality: *Neighborhood Quality Evaluation Scale* |  |  |  |  |  |  | X |  |
|  | Neighbourhood Crime: *Neighborhood Criminal Events Scale* |  |  |  |  |  |  | X |  |
|  | Family Routines: *Family Routines Inventory* |  |  |  |  |  |  | X |  |
| **Childcare Programmes** | Mother’s Participation in Parenting Programmes or Classes | X |  |  |  |  |  | X |  |
|  | Mother’s (Intentions for) Childcare Use | X |  | X | X | X | X | X | X |
|  | Mother’s (Intentions for) Childcare Type | X |  | X | X | X |  | X | X |
|  | Mother’s (Intentions for) Childcare Timing | X |  | X | X | X |  |  |  |
|  | Childcare Cost, Satisfaction, etc. |  |  |  | X | X |  | X | X |
|  | Preschool Planning |  |  |  |  |  |  | X |  |
|  | Primary School Attendance |  |  |  |  |  |  |  | X |
|  | Primary School Planning |  |  |  |  |  |  |  | X |
|  | School Readiness Traits |  |  |  |  |  |  |  | X |
| **Cognitive Assessment** | Maternal Intelligence: *Wechsler Abbreviated Scale of Intelligence (WASI)* |  | X |  |  |  |  |  |  |
| **General Questions** | Voting Behaviour | X |  | X | X | X | X | X | X |
|  | Breathing Vignettes |  |  | X |  |  |  |  |  |
|  | Age Related Questions |  |  |  | X | X | X | X | X |
|  | Relevant Notes About Interview |  |  | X | X | X | X | X | X |
| **Programme Questions** | Client Satisfaction with Programme |  |  | X | X |  | X | X |  |
|  | Frequency Mentor/IO Meetings |  |  | X | X | X | X | X | X |
|  | Contamination Questions |  |  | X | X | X | X | X | X |
| **Hopes/**  **Dreams/**  **Strengths/**  **Difficulties** | Mother’s Hopes and Dreams for New Baby | X |  |  |  |  |  |  |  |
|  | Family Problems that may Affect New Baby (Mother’s View) | X |  |  |  |  |  |  |  |
|  | Family Strengths and Qualities (Mother’s View) | X |  |  |  |  | X |  | X |
|  | Thoughts on being a Mam |  |  | X |  |  | X |  | X |
|  | Important Events Since Last Interview (Mother’s View) |  |  | X |  | X |  | X | X |
| **Sibling Behaviour** | Sibling Behaviour Questions (for 4 year old siblings only): *Newly Designed Instrument* |  |  | X |  | X |  |  |  |

**Child Survey Content**

| **Category** | **Measure/Information Obtained** | **BL** | **3** | **6** | **12** | **18** | **24** | **36** | **48** |
| --- | --- | --- | --- | --- | --- | --- | --- | --- | --- |
| **Child Development** | Difficult Temperament |  |  | X | X |  |  |  |  |
|  | Temperament: *Temperament and Atypical Behaviour Scale (TABS) Screener* |  |  |  | X |  |  |  |  |
|  | Child’s Development - Communication: *ASQ* |  |  | X | X | X | X | X | X |
|  | Child’s Development – Gross Motor: *ASQ* |  |  | X | X | X | X | X | X |
|  | Child’s Development – Fine Motor: *ASQ* |  |  | X | X | X | X | X | X |
|  | Child’s Development – Problem Solving: *ASQ* |  |  | X | X | X | X | X | X |
|  | Child’s Development – Personal-Social: *ASQ* |  |  | X | X | X | X | X | X |
|  | Child’s Development – Social-Emotional: *ASQ: SE* |  |  | X | X | X | X | X | X |
|  | Language: *MacArthur-Bates Communicative Development Inventories (CDI)* |  |  |  | X | X | X |  |  |
|  | Reading/Books |  |  |  | X |  |  |  | X |
|  | Special Services Child is Receiving |  |  |  | X | X | X | X | X |
|  | Maternal Developmental Concerns |  |  |  | X |  |  | X | X |
|  | Social Emotional Development: *Brief Infant Toddler Social and Emotional Assessment (BITSEA)* |  |  |  | X | X | X | X |  |
|  | Peer Interaction: *Experiences with Other Young Children* (from *ITSEA)* |  |  |  |  |  |  | X |  |
|  | Peer Interaction: *Peer Problems and Prosocial Behaviour* (from *SDQ)* |  |  |  |  |  |  |  | X |
|  | Cognitive Development – *Developmental Profile 3 (*Cognitive Section) |  |  |  | X | X | X | X | X |
|  | Toilet Training: *Toilet Training Q (from Developmental Milestones)* |  |  |  |  |  |  |  | X |
|  | Child Behaviour *(CBCL)* |  |  |  |  |  | X | X | X |
|  | Child Development (direct assessment): BAS III Picture Similarities |  |  |  |  |  |  |  | X |
|  | Child Development (direct assessment): BAS III Naming Vocabulary |  |  |  |  |  |  |  | X |
|  | Child Development (direct assessment): BAS III Verbal Comprehension |  |  |  |  |  |  |  | X |
|  | Child Development (direct assessment): BAS III Copying |  |  |  |  |  |  |  | X |
|  | Child Development (direct assessment): BAS III Matrices |  |  |  |  |  |  |  | X |
|  | Child Development (direct assessment): BAS III Pattern Construction |  |  |  |  |  |  |  | X |
|  | Executive Functioning & Delay of Gratification: Gift delay tasks |  |  |  |  |  |  |  | X |
| **Child Health** | Baby’s Birth Weight |  |  | X |  |  |  |  |  |
|  | Age (in days) When Baby Returned Home from Hospital |  |  | X |  |  |  |  |  |
|  | Baby’s Health Since Birth |  |  | X |  |  |  |  |  |
|  | Crying Habits/Patterns |  |  | X |  |  |  |  |  |
|  | Sleeping Habits/Patterns |  |  | X | X |  |  |  |  |
|  | Sleep: *Children’s Sleep Habits Questionnaire (CSHQ)* |  |  |  |  |  |  |  | X |
|  | Medical Visits in Last 6/12 Months |  |  | X | X | X | X | X | X |
|  | Hospital Inpatient Visits in Last 6/12 Months |  |  | X | X | X | X | X | X |
|  | Accidents and Injuries Requiring Medical Attention in Last 6/12 Months |  |  | X | X | X | X | X | X |
| **Category** | **Measure/Information Obtained** | **BL** | **3** | **6** | **12** | **18** | **24** | **36** | **48** |
| **Child Health**  ***(continued)*** | Immunizations |  |  | X | X | X |  |  |  |
|  | Breathing Difficulties |  |  | X |  |  |  |  |  |
|  | Nutrition/Eating Habits/Patterns |  |  | X | X | X | X | X | X |
|  | Child’s Current Weight (Mother Report) |  |  | X | X | X | X | X | X |
|  | Child’s Current Weight (RA Assessed) |  |  |  |  |  | X | X | X |
|  | Child’s Weight at Last Dr. Visit |  |  |  | X | X |  |  |  |
|  | Child’s Health in Last 6/12 Months |  |  |  | X | X | X | X | X |
|  | Any Diagnosed Chronic Illness |  |  |  |  |  |  | X | X |
|  | Any Diagnosed Physical Disability |  |  |  |  |  |  | X | X |
|  | Child Have Asthma |  |  |  |  |  |  |  | X |

**11. STUDY TREATMENT vigilance Safety Reporting**

**11.1 Adverse Event (AE)**

An AE is any untoward occurrence which does not necessarily have a causal relationship with the trial treatment. An AE can therefore be any unfavourable and unintended sign, symptom, or disease temporally associated with the trial treatment, whether or not considered related to the treatment.

AEs will be collected from the time of randomisation until the final 48-month follow-up visit for each participant.

**11.2 Severity of Adverse Events**

Severity of AEs will be assessed according to the following definitions:

Mild: Awareness of event but easily tolerated

Moderate: Discomfort enough to cause some interference with usual activity

Severe: Inability to carry out usual activity, including play for infants and children.

**11.3 Causality of Adverse Events**

Causality of AEs, i.e. relationship to the trial treatment, will be assessed according to the following definitions:

| Unrelated | No evidence of any causal relationship |
| --- | --- |
| Unlikely | There is little evidence to suggest there is a causal relationship (e.g. the event did not occur within a reasonable time after administration of the treatment). There is another reasonable explanation for the event (e.g. the participant’s clinical condition, other concomitant treatment). |
| Possible | There is some evidence to suggest a causal relationship (e.g. because the event occurs within a reasonable time after trial treatment). However, the influence of other factors may have contributed to the event (e.g. the participant’s clinical condition, other concomitant treatments). |
| Probable | There is evidence to suggest a causal relationship and the influence of other factors is unlikely. |
| Definite | There is clear evidence to suggest a causal relationship and other possible contributing factors can be ruled out. |

**11.4 Serious Adverse Events (SAE)**

**Definition of SAE**

An SAE is defined as any adverse event that:

- Results in death
- Is life-threatening*
- Requires hospitalisation or prolongation of existing inpatient’s hospitalisation**
- Results in persistent or significant disability or incapacity
- Is a congenital abnormality or birth defect

* “Life-threatening” in the definition of “serious” refers to an event in which the participant was at risk of death at the time of the event; it does not refer to an event which hypothetically might have caused death if it were more severe.

** “Hospitalisation” means any unexpected admission to a hospital department. It does not

apply to scheduled admissions that were planned before study inclusion or visits to an accident and emergency department (without admission).

Medical judgement should be exercised in deciding whether an adverse event/reaction is serious in other situations. Important adverse events/reactions that are not immediately life-threatening, or do not result in death or hospitalisation but may jeopardise a participant, or may require intervention to prevent one of the other outcomes listed in the definition above should also be considered serious.

**11.5 Reporting of SAEs**

Rapid reporting of all SAEs occurring during the study must be performed as detailed in SAE reporting instructions. SAEs will be reported via the eCRF within 24 hours of becoming aware of the event. All reported SAEs will be reviewed by the Chief Investigator (or designee) within 2 working days of receiving notification of the SAE report. The SAE review will be recorded on the eCRF.

SAEs will be followed up until they are resolved.

If the investigator becomes aware of safety information that appears to be related to the treatment, involving a participant who participated in the study, even after an individual participant has completed the study, this should be reported to the Sponsor.

**11.6 Definition of a Serious Adverse Reaction (SAR)**

A SAR is defined as a SAE that is judged to be related to the trial treatment.

**11.7 Definition of Unexpected and Related Serious Adverse Events**

A Related and Unexpected Serious Adverse Events is an Adverse Event that is classed as serious, is suspected to be caused by the trial treatment and is unexpected i.e. not listed as an ‘expected SAE’ in this protocol.

**11.8 Reporting of Related and Unexpected Serious Adverse Events**

All Related and Unexpected Serious Adverse Events will be notified to the Research Ethics Committee (REC) and the Sponsor within 15 days of becoming aware of the event.

Follow up of participants who have experienced a Related and Unexpected Serious Adverse Event should continue until recovery is complete or the condition has stabilised.

**11.9 Annual reporting of SAEs**

Annual safety reporting will be included in the annual progress report sent to the REC, on the anniversary of Ethics approval each year.

1. **STATISTICAL ANALYSES**

**12.1 Sample Size and Power Considerations**

The total sample size will be 300 participants (200 in the randomised high and low treatment groups and 100 in the external comparison group). Power effects (i.e. ability of a test to detect relationships) are critical in order to translate the results of the PFL evaluation into real policy effects. When a study has low power, effect size estimates will be less precise and traditional null hypothesis significance testing may incorrectly conclude that cause and effect covary. The sample size for the experimental groups was calculated based on small to moderate effect sizes (ES, standardized difference between group means) on child school readiness as identified by previous meta-analytic studies [Sweet & Appelbaum, 2004] with a power of 80% (*p* =.05, two-tailed test). As this is based on a large effect size, it is essential that recruitment and retention is maximised and attrition minimised so that the sample size is sufficiently large. As part of the evaluation, we will adopt several retention and tracking strategies.

**12.2 Data Analysis**

The primary analysis will be by intention to treat (ITT). All statistical analysis will involve comparing both treatment group A (high dosage) to treatment group B (low dosage), and groups (A+B) to control group C (external comparison group). A simple analysis of the randomised experiment will tell us if the PFL programme had a causal effect. Then, by modelling the possible relationships between the independent, mediating, and moderating variables and the outcome variable, we will investigate the antecedents and consequences of any mediating process measured, and test whether the independent variable directly, or indirectly through mediating variables, causes changes in school readiness (Shadish, Cook & Campbell, 2002). Hence causal modelling will allow us to identify the basis for any proportional improvements in outcomes. However, the reliability of both the RCT effect size and the causal modeling will critically depend on 1) power effects and 2) sample size (attrition).

**Estimation of treatment effects:** T-tests from OLS regressions on the continuous outcomes and chi-squared tests from logistic regressions on the binary outcomes will be estimated. Secondly, permutation-based hypothesis testing will be used as an alternative method of assessing the statistical significance of the observed treatment effects. Permutation testing is more suitable than standard bivariate tests, such as t-tests, as it does not depend on distributional assumptions and thus facilitates the estimation of treatment effects in small samples (Ludbrook and Dudley, 1998). A number of simulation studies have found that permutation testing has superior power advantages over parametric t tests, particularly if the data are skewed and the degree of skewness is correlated with the size of the treatment effect (e.g. Hayes, 1996). A permutation test relies on the assumption of exchangeability under the null hypothesis. If the null hypothesis is true, which implies that the programme has no impact, then taking random permutations of the treatment indicator does not change the distribution of outcomes for the high or low treatment group.

Permutation tests calculate the observed test statistic that is generated by comparing the mean outcomes of the high and low treatment groups. Next, the data are repeatedly shuffled so that the treatment assignment of some participants is switched between the groups. The *p*-value for the permutation test is computed by examining the proportion of permutations that have a test statistic more extreme than the observed test statistic. In this study, we will use permutation tests based on 100,000 replications, to estimate the programme’s impact. We will report *p*-values from one-sided tests. Effect sizes will be calculated using Cohen’s *d* for continuous variables and marginal effects (ME) for binary variables.

**Differential Attrition:** Due to differential attrition, the estimation samples at each data collection point may not be representative of the original randomized sample. This may bias the estimation of treatment effects if the type of participants who drop out of the study or do not complete a particular assessment differ across the groups. An inverse probability weighting (IPW) procedure will be applied to deal with this issue. This involves estimating logistic regression models predicting the probability of completing an interview at each assessment point by modelling attrition as a function of baseline characteristics. The probabilities generated from these logistic models will then applied as used as weights in the estimation of treatment effects (regression models and permutation testing) so that a larger weight is applied to participants that are underrepresented in the sample due to missing observations.

**Multiple Hypothesis Testing:** Analysing the impact of the programme on multiple outcome measures increases the likelihood of a Type-1 error and studies of RCTs have been criticized for overstating treatment effects due to this ‘multiplicity’ effect (Pocock et al., 1987). In order to assess the robustness of our results we will adjust the individual test p-values using the stepdown procedure, as described in Romano and Wolf (2005). The stepdown procedure involves calculating a t-statistic for each null hypothesis in a family of related outcomes and placing them in descending order. Using the permutation testing method, the largest absolute observed t-statistic is compared with the distribution of maximal permuted t-statistics. If the probability of observing this statistic by chance is high (p ≥ 0.1) we fail to reject the joint null hypothesis that the treatment has no impact on any outcome in the family being tested. If the probability of observing this t-statistic is low (p < 0.1) we reject the joint null hypothesis and proceed by excluding the most statistically significant individual hypothesis and test the subset of hypotheses that remain for joint significance. This process of dropping the most significant individual hypothesis continues until only one hypothesis remains. ‘Stepping down’ through the hypotheses allows us to isolate the hypotheses that lead to a rejection of the null. This method is superior to the Bonferroni adjustment method as it accounts for interdependence across outcomes.

A detailed statistical analysis plan will be prepared and signed off prior to any interim analyses.

1. **Patient and public involvement (PPI)**

The views of socio-economically disadvantaged parents have been considered during the development of this protocol. Parents will play a significant role in helping to conduct the study, monitor study progress and disseminate study findings.

Feedback from participants and service users has influenced the protocol in the following ways:

1. Planning to take a pragmatic approach to the delivery of the intervention, focusing on ensuring engagement with the primary caregiver, but also actively trying to involve both parents/carers if possible;
2. Assessment will be held in participants’ homes and will be flexibly timed, offering evening and weekend sessions where necessary;
3. The intervention format will be more flexible so that sessions can be held with an individual parent or with parents/carers together.

A Parent Advisory Group (PAG) will be set-up to oversee study progress throughout the duration of the trial. Members of the PAG will be asked to comment on participant information before the start of the study and will also be sent a draft version of the full study report, summary reports of the study findings for participants, and all other aspects of the dissemination strategy.

1. **REGULATORY, ETHICAL AND LEGAL ISSUES**

The study will be conducted in accordance with the Declaration of Helsinki, the Data Protection Act and the guidelines laid down by the International Conference on Harmonisation for Good Clinical Practice (ICH GCP E6 guidelines).

**14.1 Research Ethics Committee (REC) Approval**

Approval from three separate ethics committees (The UCD Human Research Ethics Committee, the Rotunda Hospital Ethics Committee, and the National Maternity Hospital Ethics Committee) will be obtained prior to the start of the trial. These approval processes will include the trial protocol, parent information sheet and consent form, questionnaires, interviews, any other written information that will be provided to the participants and any advertisements that will be used during the study.

**14.2 Approval of Amendments**

Any amendments to the protocol and information provided to participants will be submitted to the funder and the ethics committees for approval prior to implementation. An assessment of whether the amendment is substantial or non-substantial will be made prior to submitting the amendment for review. Substantial amendments may only be implemented after written ethics approval has been obtained whereas non-substantial amendments can be implemented without written approval from the ethics committee.

Amendments that are intended to eliminate an apparent immediate hazard to participants may be implemented prior to receiving funder or ethics committee approval. However, in this case, approval must be obtained as soon as possible after implementation.

**14.3 End of Trial Notification**

A notification of the end of the trial will be submitted to the ethics committee within 90 days of the final follow-up visit taking place.

**14.4 Informed Consent**

We will ensure that all participants recruited into the study are made aware of all aspects of the study using an informed consent procedure. All adult research participants (pregnant women from the catchment area) will sign and date an Informed Consent Form (ICF) before any trial procedures are performed. As described above in the ‘Recruitment’ section, eligible participants will be identified and approached by the PFL recruiter in the maternity hospital. If an eligible participant is interested in learning more about the programme, the recruiter will take her contact details. Next the recruiter will ring the eligible participant later that day to set up a recruitment appointment to take place in the village centre in the catchment area. During the recruitment appointment, the recruiter will describe the study in detail and bring the eligible participant through the information and consent form.

The information sheet will provide a full, detailed explanation of the PFL programme and the objectives of the evaluation. This information will be provided in both leaflets and oral form to each eligible participant. They will be informed about the eligibility criteria for joining the programme and why they are being invited to join the programme. The research design of the evaluation will be explicitly described, in addition to the motivation for implementing this design and the benefits of the design. In particular, the process and need for random allocation into different service groups will be emphasised, in a clear, detailed manner, with the opportunity for the eligible participants to explain their understanding of the issues and voice any questions or concerns. We will ensure that participants have a clear understanding, and acceptance, of the reasons for randomisation by holding a focus group in the pilot phase which explore respondents’ understanding, awareness, and experiences with social programmes and random allocation. It will be emphasised that there is no obligation to take part in the programme and that failure to take part will have no implications for the person’s future access to health care or social supports. Once details of the programme have been explained the eligible participant will be asked if they would like to take some time to consider joining the programme. If the eligible participant agrees to join the study she will be asked to sign the consent form. The same process will be used for all three groups.

**14.5 Patient Confidentiality**

The research team will ensure that the participant’s privacy is maintained. We will ensure that data provided by the researchers doing the fieldwork will not be identifiable to those accessing the data by adopting a number of anonymisation techniques to reduce the identification of an individual’s data. First, the names and address details will be removed and all personal identifiers will never be passed onto any third party under any circumstances. The researcher doing the fieldwork or data entry will ensure that no data collected from any individual are stored directly with contact information. Datasets will contain only uniquely assigned identification numbers, which can only be linked to specific personal details by the fieldworkers. No released or archived dataset will contain personal information. Only the principal investigators and the research assistants will have access to the identifiable data.

1. **DATA MANAGEMENT**

**15.1 Data collection**

Data collection will include direct administration of standardised interviews and child development assessments. The survey data will be collected using web-based laptops in the home setting and will take place at baseline (prior to the programme), when the child is 6, 12, 18, 24 months old and then each subsequent year of the child’s life until the start of school. These quantitative interviews will last a maximum of 2 hours. Data will be collected using Computer-Aided Personal Interviews (CAPI). Using this method, data entry, editing, and verification procedures are programmed into a laptop computer. The interviewer – or the respondent – records answers to each question using the keyboard or screen. Automatic routing ensures that there are no interviewer-driven errors in asking the correct question.

**15.2 Data management and storage**

All data collected as part of the PFL evaluation will be housed in the UCD Geary Institute. The research team will design and maintain a user-friendly internet based distributed data management application to support collection, entry, verification, validation and reporting of the data generated by the evaluation team and programme staff. The application will be integrated into a dedicated database management system (DBMS). Network security will be maintained through firewall technology, user authentication, and user roles within the database. We will develop a password protected encrypted site for sharing secure information, including protocols, manual of procedures, forms, directories, and data analysis results. Data will be stored for as long as the research is ongoing and that the same level of confidentiality guaranteed in this research will apply to the storage and use of the data in the future. Hard copies of any material linking the participant identification number to the person's contact details will be kept securely in the Investigator Site File, in a locked filing cabinet in a locked office, accessible only to key research team members.

All data will be archived at the Irish Social Science Data Archive (ISSDA) once the study has ended. The informed consent form will ask participants for their consent to archive an anonymised version of their data.

All other data including (including copies of protocols, questionnaires, correspondence, records of informed consent, and other documents pertaining to the conduct of the study) will be kept for the maximum period of time permitted by the institution. Documents will be stored in such a way that they can be accessed/data retrieved at a later date. Consideration will be given to security and environmental risks.

**15.3 Data ownership**

The UCD Geary Institute will control the data generated by the research. The data from the study is owned by the Northside Partnership (funder), however they will only have access to an anonymised version of the data with all personal identifiers and addresses omitted. No identifiable data will ever be released to a third party. Preparing for Life - Northside Partnership Group will have control of the data generated by the study.

**16. End of Trial**

The end of the trial will occur when the child of the final participant recruited has completed the last school readiness assessment in the first semester of primary school (~age 4/5 depending on when the child starts primary school).

**17. STUDY MANAGEMENT STRUCTURE**

**17.1 Project Management Group**

The Project Management group will be responsible for overseeing management of the study and operational issues. This group will meet every 2 months during the set-up phase of the trial and every 6 months thereafter. Membership will include the Principal Investigator (Orla Doyle), Co-investigators (Richard Tremblay and Sylvana Cote), and the Project Manager. The project management group will be supported by three methodological and task orientated expert panels: The **Modelling and Statistical Design** **Panel** will take responsibility for advising and directing the technical components and methodological issues of the evaluation. It will be led by **Professor Colm Harmon** (Director of the UCD Geary Institute) and Nobel Laureate **Professor James Heckman** (UCD Professor of Science and Society and Professor of Economics at the University of Chicago). The **RCT Design and Implementation Panel** will be led by **Professor Cecily Kelleher**, a clinical epidemiologist and head of the UCD School of Public Health and Health Sciences. The **Qualitative and Mixed Methods Design Panel** will be led by **Dr. Hannah Lambert**, a social psychologist at the Geary Institute with strong experience in qualitative research and mixed methodologies.

**17.2 Scientific Advisory Panel**

Supporting the Project Management Group is the ***Pritzker Consortium on Early Childhood Development***, which will serve as the Scientific Advisory Panel. The Pritzker Consortium was formed under the leadership of James Heckman and includes leading scientists who had conducted RCTs in the field of youth interventions. The Pritzker Consortium has received a major 5-year research award from the National Institute of Child Health and Human Development to conduct innovative and theory-guided data analyses of the leading RCT data sets involving young children. The members of the Consortium have direct experience of the initiatives in Ireland developed and funded by the Atlantic Philanthropies DCY programme and have extensive experience in designing and overseeing the randomisation of children, families, schools, and/or communities to alternative treatment conditions, and ensuring highly ethical conduct. Examples of early childhood interventions which members of the Pritzker has developed and worked on include the Abecedarian Project, Perry Preschool Program, High/Scope Pre-School Curriculum Study, Chicago Child-Parent Center, the Infant Health and Development Program, and Project CARE. The Pritzker members have written extensively about methods related to design, conduct, and analysis of RCTs and will share this expertise in the planning, conduct and analysis of the evaluation of the PFL Programme. Two of the leading international experts - Professors Craig and Sharon Ramey (Georgetown University) - will represent this Consortium for the PFL evaluation and will continue to meet regularly with the project management team.

**17.3 Data Monitoring and Ethics Committee (DMEC)**

An independent Data Monitoring and Ethics Committee (DMEC) will be established to oversee safety of the trial. The DMEC will review SAE reports and key data as required. The DMEC will develop, in agreement with the investigators and TSC, a charter outlining their responsibilities, planned interim analyses and operational details. The DMEC will meet prior to the start of the trial to agree the charter.

**17.4 Publication policy**

The results from the trial will be submitted for publication in a peer-reviewed journal irrespective of the outcome. The Project Management Group will be responsible for approval of the main manuscript prior to submission for publication. At the end of the study, children’s parents will be able to request a copy of the results of the study from the investigator at that site.

**REFERENCES**

Brooks-Gunn, J. (2003), Do you believe in magic? *Social Policy Report*, 17(1), 3-16.

Carneiro, P. and J. J. Heckman (2003), Human Capital Policy. In J. Heckman, A. Krueger (Eds.), *Inequality in America: What Role for Human Capital Policy?* (pp. 77-240). Cambridge, Mass.: MIT Press.

Census. (2006). Retrieved from <http://beyond2020.cso.ie/census/ReportFolders/ReportFolders.aspx>

Clarke, A.T., & Kurtz-Costes, B. (1997). Television viewing, educational quality of the home environment, and school readiness. *The Journal of Educational Research*, 90, 279-285.

Costeff, H., & Kulikowski, Z. (1996). The developmental profile of disadvantaged six-year-old children. *The British Journal of Developmental Disabilities*, 62, 45-53.

Duncan, G. J. and J. Brooks-Gunn (1997), *Consequences of growing up poor*. New York: Russell Sage Foundation.

Geoffroy, M.-C., **Côté, S.M.**, Borge, A., Larouche, F., Séguin, J. R. & Rutter, M. (In press). Association between nonmaternal care in the first year of life and children's receptive language skills prior to school entry: The moderating role of the socioeconomic status. *Journal of Child Psychology and Psychiatry*.

Halfon, N., E. Shulman and M. Hochstein (2001), “Brain development in early childhood”. In: N. Halfon, E. Shulman, M. Hochstein (Eds.), *Building Community Systems for Young Children*. UCLA Center for Healthier Children, Families and Communities.

Hayes, A. (1996). Permutation test is not distribution-free: Testing h0 :) = 0. *Psychological Methods,* 1, 184–198.

Horvat, L., & Svetina, M. (1993). Effects of social-educational family status on child’s school success in first grade. *Studia Psychologica*, 35, 4-5.

Janus, M., and E. Duku (2007), The school entry gap: Socioeconomic, family, and health factors associated with children's school readiness to learn. *Early Education & Development*, 18(3), 375-403.

Karoly, L. A., M. R. Kilburn and J. S. Cannon (2005), *Early childhood interventions:*

Ketterlinus, R.D., Henderson, S., Lamb, M.E. (1991). The effects of maternal age-at-birth on children’s cognitive development. *Journal of Research on Adolescence*, 1, 173-188.

Kinard, E.M., & Reinherz, H. (1987). School aptitude and achievement in children of adolescent mothers. *Journal of Youth and Adolescence*, 16, 69-87.

Ludbrook, J., Dudley, H. (1998). Why permutation tests are superior to t and F tests in biomedical research. The American Statistician 52, 127-32.

McLoyd, V. (1998), “Socioeconomic disadvantage and child development”, *American Psychologist*, 53(2), 185-204.

Najman, J. M., R. Aird, W. Bor, M. O’Callaghan, G. M. Willians and G. J. Shuttlewood (2004), The generational transmission of socioeconomic inequalities in child cognitive development and emotional health, *Social Science and Medicine*, 58, 1147–1158.

Olds, D. L., C. R. Henderson, H. Kitzman, J. J. Eckenrode, R. E. Cole and R. C Tatelbaum (1999), Prenatal and infancy home visitation by nurses: Recent findings, *The Future of Children, 9*, 44-65. *Proven results, future promise*. Santa Monica, CA: RAND Corporation.

Pocock, S. J., Hughes, M. D., Lee, R. J. (1987). Statistical problems in the reporting of clinical trials. A survey of three medical journals. New England Journal of Medicine 317, 426-32.

Raver, C. (2003), “Young children’s emotional development and school readiness”, *ERIC Digest*.

Ricciuti, H.N. (1999). Single parenthood and school readiness in White, Black, and Hispanic six and seven-year-olds. *Journal of Family Psychology*, 13, 450-465.

Romano, J.P., Wolf, M. (2005). Exact and approximate stepdown methods for multiple hypothesis testing. Journal of American Statistical Association 100, 94-108.

Rouse, C., J. Brooks-Gunn and S. McLanahan (2005), School readiness: Closing racial and ethnic gaps, *The Future of Children*, 15(1), 5-15.

Shadish W, Cook T, and Campbell D. (2002) *Experimental and Quasi-Experimental designs for Generalized Causal Inference*. Boston: Houghton Mifflin Company.

Shonkoff, J. P., and D. A. Phillips (2000), National Research Council and Institute of Medicine. *From Neurons to neighbourhoods: The Science of Early Childhood Development*. Report of the Committee on Integrating the Science of Early Childhood Development. Washington DC: National Academy press.

Stipek, D. J., and R. H. Ryan (1997), Economically disadvantaged preschoolers: Ready to learn but further to go, *Developmental Psychology*, 33(4), 711-723.

Sweet, M. A., and M. I. Appelbaum (2004), “Is home visiting an effective strategy? A meta-analytic review of home visiting programs for families with young children”, *Child Development*, 75, 1435-1456.
