# Supplementary material for: Can Early Intervention Improve Maternal Well-Being? Evidence from a Randomized Controlled Trial
Source: PLoS One. 2017 Jan 17;12(1):e0169829. doi: 10.1371/journal.pone.0169829 (PMC5241149; doi:10.1371/journal.pone.0169829)
Supplement: S1 Table — (DOCX) [file pone.0169829.s004.docx]

**S1 Table** *Descriptive statistics*

|  | *Baseline Interview* | | | |
| --- | --- | --- | --- | --- |
|  | N ^a^  (*n*_TREAT_*/ n*_CONTROL)_ | *M*_TREAT_  (*SD*) | *M*_CONTROL_  (*SD*) | *p*-value |
|  |  |  |  |  |
| Maternal Age | 101  (46/55) | 26.00  (5.45) | 25.35  (5.75) | 0.56 |
| Child gender: Male | 101  (46/55) | 0.48  (0.51) | 0.31  (0.47) | 0.08* |
| Number of non-PFL children | 101  (46/55) | 1.00  (1.32) | 1.05  (1.25) | 0.83 |
| First time mother | 101  (46/55) | 0.50  (0.51) | 0.47  (0.50) | 0.79 |
| Lives in public housing | 101  (46/55) | 0.59  (0.50) | 0.55  (0.50) | 0.68 |
| Married | 101  (46/55) | 0.17  (0.38) | 0.16  (0.37) | 0.89 |
| Maternal Work Status |  |  |  |  |
| Employed | 101  (46/55) | 0.39 (0.49) | 0.36  (0.49) | 0.78 |
| Looking after family | 101  (46/55) | 0.13  (0.34) | 0.13  (0.34) | 0.96 |
| Unemployed | 101  (46/55) | 0.43  (0.50) | 0.40  (0.50) | 0.73 |
| Other | 101  (46/55) | 0.04 (0.21) | 0.11  (0.31) | 0.23 |
| Maternal Education |  |  |  |  |
| Lower than second level education | 101  (46/55) | 0.41 (0.50) | 0.44  (0.50) | 0.82 |
| Second level education | 101  (46/55) | 0.20 (0.40) | 0.25  (0.44) | 0.49 |
| Primary degree/non-degree qualification | 101  (46/55) | 0.39 (0.49) | 0.31  (0.47) | 0.39 |

*Notes.* **‘**N’ indicates the sample size. ‘M’ indicates the mean. ‘SD’ indicates the standard deviation. The *p*-values based are on a test of differences between the treatment and control groups. ^a^ One participant did not complete a baseline interview.

***** Significant at the 1 percent level.

**** Significant at the 5 percent level.

*** Significant at the 10 percent level.
