# Supplementary material for: Can Early Intervention Improve Maternal Well-Being? Evidence from a Randomized Controlled Trial
Source: PLoS One. 2017 Jan 17;12(1):e0169829. doi: 10.1371/journal.pone.0169829 (PMC5241149; doi:10.1371/journal.pone.0169829)
Supplement: S2 Table — (DOCX) [file pone.0169829.s005.docx]

**S2 Table** *Pairwise Correlations between Well-being Measures*

|  | Net Affect | Positive Affect | Negative Affect | U-Index | Positive Mood Yesterday | Life Satisfaction | PSI Total Stress |
| --- | --- | --- | --- | --- | --- | --- | --- |
| Net Affect | 1 | - | - | - | - | - | - |
| Positive Affect | 0.85*** | 1 | - | - | - | - | - |
| Negative Affect | -0.75*** | -0.28*** | 1 | - | - | - | - |
| U-Index | -0.71*** | -0.40*** | 0.79*** | 1 | - | - | - |
| Positive Mood | 0.28*** | 0.22** | -0.41*** | -0.25** | 1 | - | - |
| Life Satisfaction | 0.13 | 0.03 | -0.20* | -0.10 | 0.06 | 1 | - |
| PSI Total Stress | -0.34*** | -0.34*** | 0.20* | 0.08 | -0.38*** | -0.19* | 1 |

**Notes:** The pairwise correlations are calculated at the individual level. For Life Satisfaction the original four category variable is used to calculate the correlation coefficient rather than the two category outcome variable.

***** Significant at the 1 percent level.

**** Significant at the 5 percent level.

*** Significant at the 10 percent level.
